# Antioxidant Activity and Mechanisms of Action of Natural Compounds Isolated from Lichens: A Systematic Review

**DOI:** 10.3390/molecules190914496

**Published:** 2014-09-12

**Authors:** Pollyanna A. S. White, Rita C. M. Oliveira, Aldeidia P. Oliveira, Mairim R. Serafini, Adriano A. S. Araújo, Daniel P. Gelain, Jose C. F. Moreira, Jackson R. G. S. Almeida, Jullyana S. S. Quintans, Lucindo J. Quintans-Junior, Marcio R. V. Santos

**Affiliations:** 1 Department of Physiology, Federal University of Sergipe, São Cristóvão, Sergipe 49100-000, Brazil; E-Mails: jullyanaquintans@gmail.com (J.S.S.Q.); lucindojr@gmail.com (L.J.Q.-J.); 2 Medicinal Plants Research Center, Federal University of Piauí, Teresina, Piauí 64049-550, Brazil; E-Mail: menesesoliveira@gmail.com; 3 Department of Biophysic and Physiology, Federal University of Piauí, Teresina, Piauí 64049-550, Brazil; E-Mail: aldeidia@gmail.com; 4 Nucleus of Pharmacy, Federal University of Sergipe, Lagarto, Sergipe 49100-000, Brazil; E-Mail: maiserafini@hotmail.com; 5 Department of Pharmacy, Federal University of Sergipe, São Cristóvão, Sergipe 49100-000, Brazil; E-Mail: adriasa2001@yahoo.com.br; 6 Department of Biochemistry, Federal University of Rio Grande do Sul, Porto Alegre, Rio Grande do Sul 90035-003, Brazil; E-Mails: dgelain@yahoo.com.br (D.P.G.); jcfm@ufrgs.br (J.C.F.M.); 7 Center for Studies and Research of Medicinal Plants, Federal University of San Francisco Valley, Petrolina, Pernambuco 56304-205, Brazil; E-Mail: jackson.guedes@univasf.edu.br

**Keywords:** lichens, antioxidants, antioxidant response elements, DPPH, cancer, chronic disease

## Abstract

Chronic diseases such as cancer, diabetes, neurodegenerative and cardiovascular diseases are characterized by an enhanced state of oxidative stress, which may result from the overproduction of reactive species and/or a decrease in antioxidant defenses. The search for new chemical entities with antioxidant profile is still thus an emerging field on ongoing interest. Due to the lack of reviews concerning the antioxidant activity of lichen-derived natural compounds, we performed a review of the antioxidant potential and mechanisms of action of natural compounds isolated from lichens. The search terms “lichens”, “antioxidants” and “antioxidant response elements” were used to retrieve articles in LILACS, PubMed and Web of Science published until February 2014. From a total of 319 articles surveyed, 32 met the established inclusion and exclusion criteria. It was observed that the most common isolated compound studied was usnic acid, cited in 14 out of the 32 articles. The most often described antioxidant assays for the study of *in vitro* antioxidant activity were mainly DPPH, LPO and SOD. The most suggested mechanisms of action were scavenging of reactive species, enzymatic activation and inhibition of iNOS. Thus, compounds isolated from lichens are possible candidates for the management of oxidative stress, and may be useful in the treatment of chronic diseases.

## 1. Introduction

Oxidative stress is characterized as an imbalance between the production of reactive species and antioxidant defense activity, and its enhanced state has been associated with many of the chronic diseases such as cancer, diabetes, neurodegenerative and cardiovascular diseases [1]. Based on that, many research groups have driven efforts to assess the antioxidant properties of natural products. These properties have been investigated through either chemical (*in vitro*) or biological (*in vivo*) methods, or both [2]. The results of these researches have led some to suggest that the long-term consumption of food rich in antioxidants can retard or avoid the ocurrence of such diseases [3,4].

According to Brewer [5], the effectiveness of a large number of antioxidant agents is generally proportional to the number of hydroxyl (OH) groups present in their aromatic ring(s). Based on that, the natural compounds would seem to have better antioxidant activity than the currently used synthetic antioxidants, making them a particularly attractive ingredient for commercial foods [5].

Despite the large number of natural products that are currently consumed as antioxidant agents, the search for new chemical entities with antioxidant activity still remains a burgeoning field. In this context, the lichens have played an important role as a source for new antioxidant agents.

Lichens are symbiotic organisms consisting of a fungus and one or more photosynthetic partners, the latter usually being either a green alga or a cyanobacterium [6,7]. They are found in a wide variety of natural habitats or in places with low temperatures, prolonged darkness, drought and continuous light [8]. Lichens produce characteristic and unique secondary metabolites, and most of them occur exclusively in these symbiotic organisms [9]. The most common lichen compounds are aromatic polyketides, particularly depsides, depsidones, depsones, dibenzofurans, and chromones [10].

Lichens have been used in the folk medicine for numerous purposes, among them as astringents, laxatives, anticonvulsives, antiemetics, antiasthmatics, anti-inflammatories, antibiotics, and also for the treatment of cardiovascular, respiratory, and gastric disorders [11]. Furthermore, pharmacological and biotechnological studies have been carried out in order to test and to develop biomaterials containing lichen-isolated natural compounds for humans use [12,13].

Therefore, based on that, and also due to the lack of reviews concerning the antioxidant activity of lichen-isolated natural compounds, we have performed, for the first time, a systematic review of the literature that provides an overview of the antioxidant properties and mechanisms of action of natural compounds isolated from lichens.

## 2. Results and Discussion

**Figure 1:**
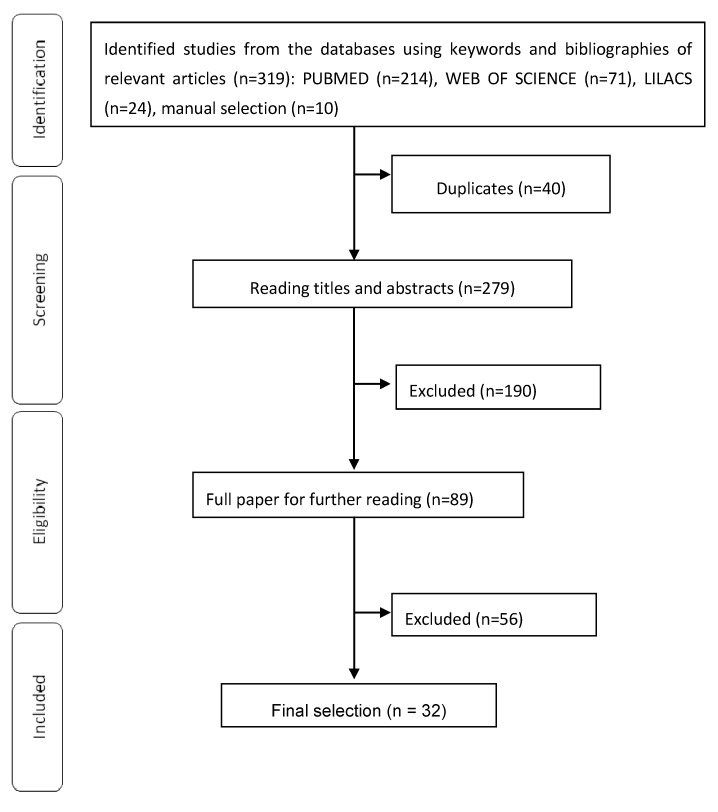
Flowchart of included studies. Studies were excluded according to the following exclusion criteria: studies in humans, studies of mixtures of substances or extracts from lichens, review articles, meta-analyses, abstracts, conference proceedings, editorials/letters, case reports.

A total of 319 abstracts/citations was identified for preliminary review from electronic and manual searches. The primary search identified 319 articles, with 214 from PubMed, 24 from LILACS, 71 from Web of Science and 10 from manual selection. After the removal of duplicates and screening for relevant titles and abstracts, a total of 89 articles was submitted for a full-text review. Thirty two articles met the inclusion and exclusion criteria established. A flow chart illustrating the progress of study selection and number of articles at each stage were performed as described in Barreto *et al*. [14] (Figure 1). The characteristics of included studies were summarized in the Table 1.

The 32 studies were developed between 2000 and 2014, in 15 different countries, being eight from Asia, seventeen from Europe and six from South America. Among them, Serbia was the country with the largest number of studies, represented by six studies, followed by Brazil with five. Among these articles, it was observed that the most common isolated compound studied was usnic acid, which was cited in 14 of the total 32 articles. Besides usnic acid, another nine compounds were frequently referred to: atranorin, diffractaic acid, lecanoric acid, lobaric acid, stictic acid, salazinic acid, fumarprotocetraric acid, physodic acid and the orsellinates.

### 2.1. Usnic Acid

**Figure 2:**
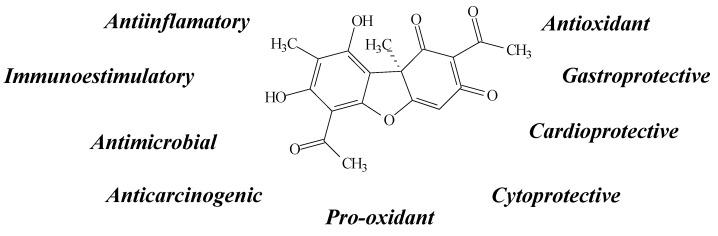
Usnic acid structure and biological activities.

Usnic acid (UA) is one of the most common and abundant lichen metabolites. It belongs to the dibenzofuran family and can be isolated from *Usnea longissima*, *U. articulate*, *U. complanata*, *U. meridionalis*, *U. barbata* and *Cladonia arbuscula*, among other species [15,16,17,18,19,20]. Several biological properties have been observed from this compound, such as gastroprotective [15], cardiovascular [17] and cytoprotective [15,21], immunoestimulatory [18], antimicrobial [19], anti-inflammatory [22] and anticarcinogenic activities [19,23,24,25], mostly through its antioxidant acttion in reducing oxidative damage [17,25,26,27] (Figure 2).

According to Odabasoglu *et al.* [15], UA (25, 50, 100, and 200 mg/kg) exerted gastroprotective effects on indomethacin-induced gastric ulcers in rat, by reducing oxidative damage. UA promoted the increase of superoxide dismutase activity (SOD), glutathione peroxidase activity (GPx), total glutathione (GSH) and constitutive nitric oxide synthase (cNOS) activities, and through the reduction of catalase (CAT), glutathione reductase (GR), lipid peroxidation (LPO), inducible nitric oxide synthase (iNOS) and myeloperoxidase (MPx) activities. Behera, Mahadik, and Morey [16], studying the cardiovascular protective activity of UA (0.005–0.2 mg/mL), observed moderate to strong antioxidant activity, in a concentration-dependent manner, in the free radical scavenging assay (FRSA), nitric oxide radical scavenging assay (NOR) and lipid peroxidation assay (LPO). Stronger scavenging activity was likewise verified by Ranković *et al.* [19] in the DPPH, reducing power and SAS assays. In this same study, a very strong antimicrobial activity was also detected against bacteria and fungi (*B. mycoides*, *B. subtilis*, *E. coli*, *K. pneumonia*, *S. aureus*, *A. flavus*, *A. fumigatus*, *C. albicans*, *P. purpurescens*, *P. verrucosum*) in the MIC assay.

Jin, Li and He [22], studying the molecular mechanisms responsible for the anti-inflammatory effects of UA (1, 2.5, 5, 10, and 20 µM), showed that this compound presented a dose-dependent inhibitory effect on lipopolysaccharide (LPS)-induced tumor necrosis factor–α (TNF-α) and nitric oxide (NO) production in macrophages RAW 264.7. This effect could be associated with decreased synthesis of TNF-α mRNA and inducible nitric oxide synthase (iNOS) protein [22].

Strong cytotoxic action of UA (25–100 µM) was demonstrated in the 3-[4,5-dimethylthiazol-2-yl]-2,5 diphenyltetrazolium bromide (MTT) assay, against several human cancer cell lines such as FemX (melanoma), LS174 (colon carcinoma) [19], MCF-7 (breast adenocarcinoma), HeLa (cervix adenocarcinoma), HCT-116 (colon carcinoma) [23], U937 (monocytic leukemia), HL-60 (monocytic leukemia) [24], A2780 (ovarian carcinoma), SK-BR-3 (breast adenocarcinoma), HT-29 (colon adenocarcinoma), HCT-116 p53^−/−^ (colon carcinoma p53-null subline) and Jurkat (T cells lymphocyte leukaemia) [25]. This action can be determined by pro-apoptotic activity, supported by the suppression of viability and cell proliferation, that correlated more strongly with an increased number of floating cells. Moreover, cell cycle distribution can present a variation, revealing an accumulation of cells in S-phase [25]. Nevertheless, Ranković *et al.* [19], observed pro-apoptotic effects correlated with an increase in the number of cells in the sub-G_1_ phase, while the percentage of cells in the S-phase and G_2_/M phase remained unchanged compared to the controls, supporting a G_1_ phase arrest mechanism. These results provide scientific data supporting potential use of UA in the treatment of several types of cancer.

On the other hand, protective effects were found by De Paz *et al.* [21] against hydrogen peroxide-induced damage in U373 MG cells (human glioblastoma astrocytoma). UA showed a strong antioxidant capacity in the oxygen radical absorbance capacity (ORAC) assay, indicating significantly reduced radical oxygen species (ROS) production. These data indicate that UA (5–50 µg/mL) could act as an antioxidant agent against neurodegenerative disorders associated with oxidative damage, such as Alzheimer’s and Parkinson’s disease. In a study by Santos *et al.* [18], usnic acid induced the greatest release of NO in peritoneal macrophages, promoting a immunostimulatory effect.

Polat *et al.* [26], assessing the genotoxic and antioxidant effects of UA in human blood cells, observed that UA did not induce mutagenic effects on human lymphocytes, and increased total antioxidant capacity (TAC) at low doses (1 and 5 µg/mL) and in total oxidative status (TOS), at a high dose (200 µg/mL). However, at this high dose, UA significantly decreased TAC levels.

Conversely, no antioxidant action of UA was observed by Dévéhat *et al.* [16] and Thadhani *et al.* [7] on the DPPH assay. The radical-scavenging effect of antioxidants on DPPH is a simple and reliable method to quantify the hydrogen donating potency of chemicals. Since no activity of UA was observed in the DPPH, it does not seem to have labile hydrogen atoms. As for the contradictory data in the LPO assay, the different concentrations utilized could have influenced the test results.

Furthermore, in a study conducted by Rabelo *et al.* [27], who tested the UA redox properties against different reactive species (RS) generated *in vitro*, and evaluated its action on SH-SY5Y neuronal-like cells upon hydrogen peroxide (H_2_O_2_) exposure, it was observed that UA could display significant antioxidant properties in the TRAP/TAR and OH radical scavenging activity tests. It also induced cell detachment and loss of viability of SH-SY5Y cells at higher concentrations (20 µg/mL) alone or in the presence of H_2_O_2_ or 1% of FBS, related to the increase of intracellular ROS, inducing an oxidative stress scenario, potentiated in the presence of H_2_O_2_. The pro-oxidant properties in biological systems might be responsible for the potential neurotoxicological effects of UA.

The heterocyclic structure composed by conjugated dienes and polar OH groups of UA suggests that this molecule is able to act as a redox-active agent, thus interacting with different RS as observed in some *in vitro* assays by the works described above. Nonetheless, the results observed in different biological assays indicate that UA may exert either pro-oxidant or antioxidant effects in different cell types and tissues, thus other mechanisms such as modulation of antioxidant enzymes and cell detoxification systems must be further investigated to address the mechanism of its redox actions. Also, UA may influence the polarity of the inner mitochondrial membrane [20], which may be reflected in changes in basal RS production to varying degrees in different cell types. As the profile of mitochondria expression and activity varies according the cell type, the effect of UA on mitochondrial integrity and activity should be further investigated in the different cell models studied.

### 2.2. Atranorin

**Figure 3:**
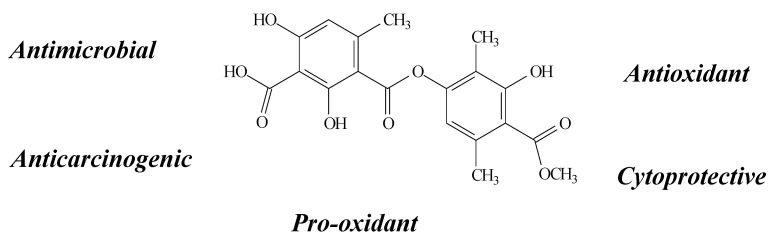
Atranorin structure and biological activities.

Atranorin (Figure 3) is an important member of the depside group, found in a variety of lichen species, among them *Cladina kalbii*, *C. furcata, Lethariella canariensis, Hypotrachyna revoluta* and *Usnea articulata* [8,16,24,28,29]. It also possesses several biological properties such as antimicrobial [29], anticarcinogenic [24,25,28], cytoprotective [8], antioxidant [8,28,29,30] and pro-oxidant activity [8].

Kosanić *et al.* [28] found that atranorin (4 to 0.00181 mg/mL) presented a very strong antimicrobial activity against bacteria and fungi (*B. mycoides*, *B. subtilis*, *E. coli*, *K. pneumonia*, *S. aureus*, *A. flavus*, *A. fumigatus*, *C. albicans*, *P. purpurescens*, *P. verrucosum*) in the MIC assay. Corroborating with this data, Marante *et al.* [24] observed moderate antibacterial activity (in the 0.25–512 µg/mL concentration range), however, only against *S. aureus*.

Stronger cytotoxic activity was verified by atranorin, in the MTT assay, in several human cancer cell lines such as U937, HL-60 [24], FemX, LS174 [28], A2780, MCF-7, SK-BR-3, HT-29, HCT-116 p53^−/−^, HCT-116 p53^+/+^ and Jurkat [25]. In the study conducted by Kosanić *et al.* [28], the antiproliferative activity was accompanied by a stronger increase in the percentage of the sub-G_1_ population and concomitant decrease in G_2_/M, leading to a G_0_/G_1_ cell cycle block and inducing apoptosis in a cell cycle-dependent manner. Bačrová *et al.* [25] also identified higher pro-apoptotic activity (except on the A2780 cell line), supported by the inhibition of clonogenic ability and cell proliferation. On the other hand, Melo *et al.* [8] demonstrated no cytotoxic effect on the SH-SY5Y cells, bestowing atranorin with the capacity to induce cytoprotection in the presence of toxic concentrations of H_2_O_2_.

A peroxyl radical scavenging effect of atranorin (0.1 to 100 µg/mL) in TRAP/TAR assays was also observed by Melo *et al.* [8]. It also presented a significant superoxide dismutase-like activity, evidencing an antioxidant potential against superoxide radicals. Conversely, it presented a pro-oxidant capacity in a lipid-rich system, enhancing TBARS formation induced by thermolysis of 2,20-azo-bis-(2-amidinopropane) dihydrochloride (AAPH) incubation. In assays where the antioxidant potential against NO and H_2_O_2_ was evaluated, atranorin was also shown to enhance the production of such species, acting as a pro-oxidant molecule, but only at higher concentrations.

Hydrogen peroxide is known to induce cell death by oxidative stress-dependent necrosis and apoptosis, which results from severe oxidative damage to DNA, lipids and proteins. It is very likely that the pro-oxidative observed by Melo *et al.* [8] is related to the concentration range tested, since in the study of Marante *et al.* [24] lower doses (100–250 µM) acted reducing H_2_O_2_/FeCl_2_ and inhibiting LPO, protecting from oxidative damage by the inhibition of both ROS and free radicals [24]. These last findings were corroborated by Papadopoulou *et al.* [29] and Kosanić *et al.* [28], whom also observed a noteworthy antioxidant activity of atranorin (0.012–0.017 mg/mL) on the Co(II)/EDTA-induced luminol plateau chemiluminescence assay and very strong antioxidant activity in the DPPH, SAS and reducing power assays, respectively. These antioxidant properties could be contributing to its pharmacological effects, such as to reduce the damage effects on skin and to modulate the healing process of wounds [30].

Although antioxidant effects were described by Melo *et al.* [8], Marante *et al.* [24], Kosanić *et al.* [28] and Papadopoulou *et al.* [29], neither Dévéhat *et al.* [16] nor Thadhani *et al.* [7] found any antioxidant activity of atranorin on SOR and SAS, respectively. The presence of at least one free OH group, attached to either ring A or B, is necessary for the SOR activity; atranorin has a deactivating aldehyde group at C-3 of ring A [7]. The quantity of free OH might also be involved in the superoxide scavenging-effect [5]. Other similarly substituted compounds should be tested to determine whether these substituents are associated with the activity.

### 2.3. Lecanoric Acid

**Figure 4:**
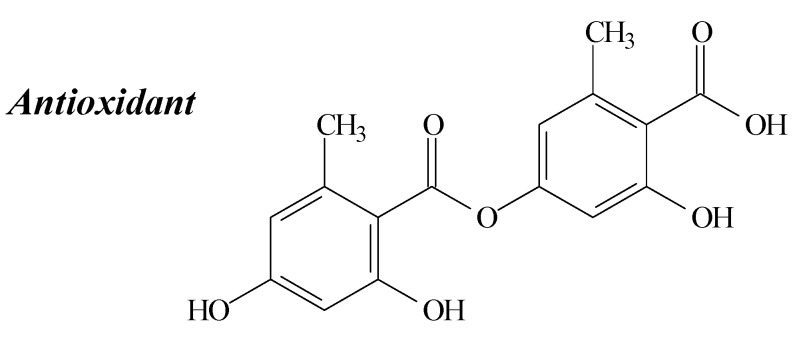
Lecanoric acid structure and biological activities.

Lecanoric acid (Figure 4) belongs to the depsidone family and can be isolated from several lichens, including *Usnea subvacata* Motyka, *Parmotrema stuppuem*, *Parmotrema tinctorum* and *Parmotrema grayana* [18,28,31,32].

Research conducted by Thadhani *et al.* [7], demonstrated a high SOR activity of lecanoric acid, comparable to that of the standards propyl gallate (PG) and butylated hydroxyanisole (BHA). They also verified a moderate activity in NOR and DPPH assays, corroborating the report of Lopes *et al.* [32]. Jayaprakasha and Rao [31] also observed moderate antioxidant potential in the β-carotene-linoleate model system. The moderate antioxidant activity of lecanoric acid could be justified by its electron-attracting properties due to the two hydrogen bonds between the 2'-OH and 1'- COOCH_3_/COOH groups and the 2-OH and 1-COO- groups and also due to the presence of the COO- group, conjugated with an aromatic ring, which is also electron-attracting [31]. Anyhow, these studies suggest that lecanoric acid is potentially able to interact with superoxide radicals.

### 2.4. Diffractaic Acid

**Figure 5:**
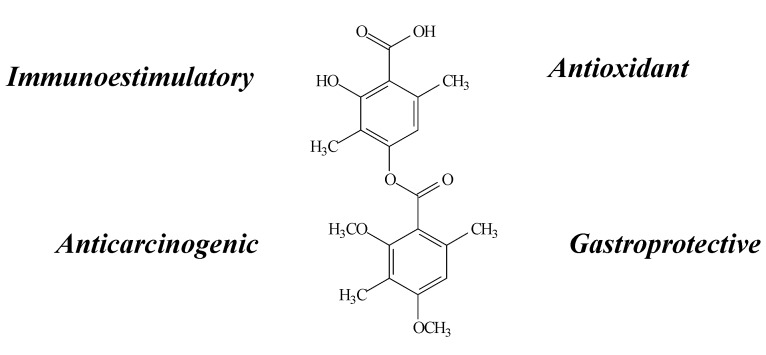
Diffractaic acid structure and biological activities.

Diffractaic acid (Figure 5) is part of the depside group and can be isolated from *Usnea longissima*, *U. subvacata* Motyka and *Protousnea magellanica* (Mont.) Krog [18,23,33,34]. Among its properties, antioxidant [35], gastroprotective [35], immunoestimulatory [18] and anticarcinogenic effects [23,36] can be observed.

Research conducted by Bayir *et al.* [33] verified that different doses of diffractaic acid (25, 50, 100, and 200 mg/kg) decreased MPx and iNOS and increased cNOS activities, suggesting that it could play an inhibitory role in neutrophil infiltration into gastric mucosal tissues, resulting in a gastroprotective effect on indomethacin-induced gastric lesions in rats. It also increased levels of SOD, GPx, GSH and decreased the effect on LPO, indicating an enhancing effect on the antioxidant defense system against oxidative tissue damage. It is not known, however, whether these effects resulted from transcriptional or post-translational effects on these enzymes. Molecules presenting polyphenolic structures may influence the oxidation state of intracellular thiol groups, thus affecting the activation of redox-sensitive transcription factors such as Nrf2, which modulates the transcription of genes involved in the antioxidant response [34,36].

In the study of Odabasoglu *et al.* [36], it was demonstrated that oral treatment of rabbits with diffractaic acid (30 mg/kg), in olive oil, can exert pro-apoptotic effects in tissue surrounding titanium implants, through the activation of initiator caspases (caspases 2, 8, and 9), executioner caspase (caspase 3), SOD activity and GSH levels, and the inhibition of the enzymatic activities of iNOS and MPx. These data indicate that this could be a possible mechanism involved in the protection towards cancer development in several tissues, by means of natural chemically induced apoptosis. The modifications on the levels of endogenous antioxidants and enzymes involved in the antioxidant response along with the activation of initiator caspases also suggest that the effect of diffractaic acid on mitochondrial homeostasis should be investigated in detail.

Santos *et al.* [18], on the other way, observed a high activity of diffractaic acid in the release of NO in mice macrophage cells, which could contribute to the production and extracellular release of ROS, bringing forth immunostimulatory effects.

Brisdelli *et al.* [23] demonstrated that diffractaic acid (2.5–100 µM) presents good antiproliferative activity against HCT-116 cells and reduction of viability in MCF-7 and HeLa cells, through the MTT assay. However, no free radical scavenging activity was observed, and the lichen metabolites did not significantly increase the intracellular ROS level and did not prevent oxidative injury induced by *t*-butyl hydroperoxide in HeLa cells. These findings suggest that the cytotoxic effect was not triggered by reactive oxygen overproduction, but could be related to diffractaic acid ability to induce programmed cell death through a caspase-dependent pathway.

### 2.5. Lobaric Acid

**Figure 6:**
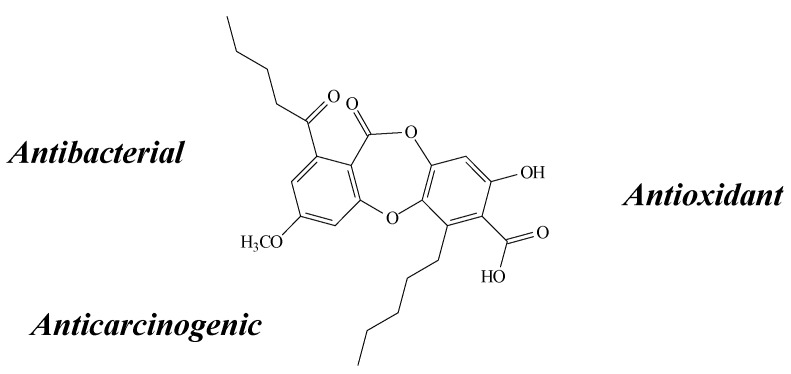
Lobaric acid structure and biological activities.

Lobaric acid (Figure 6) is a member of the depsidone family and can be isolated from several Antarctic lichens, including *Sterocaulon alpinum* e *Cladonia* sp. [7,37].

In the study of Bhattarai *et al.* [37], this compound (0–4000 µM) was shown to have antibacterial activity against Gram-positive bacteria *Staphylococcus aureus* and *Bacillus subtilis*, through a MIC assay. Nevertheess, moderate antioxidant activity by reducing DPPH free radicals in a dose-dependent manner without any toxic effects was also observed [37].

Brisdelli *et al.* [23] identified lobaric acid effects on cytotoxic activity against HeLa and HCT cells lines only at higher concentrations (100 µM) on an MTT assay. However, no free radical scavenging activity was observed in the DPPH assay and no increase in cellular ROS, suggesting that cytotoxic action was not triggered by ROS formation. These results are in accordance with previous research conducted by Thadhani *et al.* [7], in which no activity on DPPH and NOR was observed.

Although no dose or concentration was specified by Thadhani *et al.* [7], the contradictory responses of lobaric acid on the DPPH assay between Bhattarai *et al.* [37] and Brisdelli *et al.* [22] could be related to the concentration range, since Bhattarai *et al.* [37] used much higher doses than Brisdelli *et al.* [22] and the DPPH activity was found to be dose-dependent.

### 2.6. Stictic Acid

**Figure 7:**
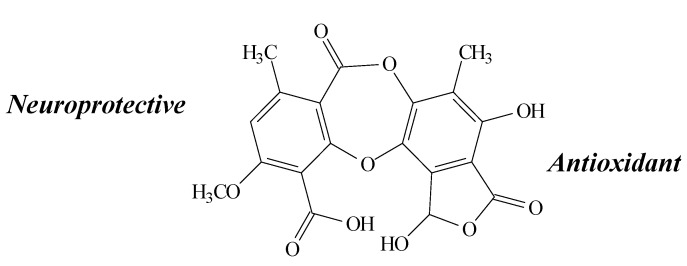
Stictic acid structure and biological activities.

Stictic acid (Figure 7) is a β-orcinol depsidone [21]. It can be isolated from *Usnea articulata*, *Xanthoparmelia conspersa*, *Xanthoparmelia camtschadalis* and *Ypotrachyna revoluta* [16,21,29].

In the study of De Paz *et al.* [21], stictic acid showed protective effects against U373MG cell line (5 and 10 µg/mL) by decreasing ROS production induced by hydrogen peroxide in an ORAC assay, inducing neuroprotection through their antioxidant activity. It also presented a noteworthy antioxidant activity (concentration range 0.012–0.017 mg/mL) according to the radical scavenging Co(II)/EDTA-induced luminol plateau chemiluminescence assay [29]. However, no antioxidant activity was demonstrated by Dévéhat *et al.* [26] (at 187.5–3000 µM) through the DPPH and SAS assays. A possible explanation for this is the molecular conformation, which could be affecting their antiradical activity, intrinsically related to DPPH.

### 2.7. Fumarprotocetraric Acid

**Figure 8:**
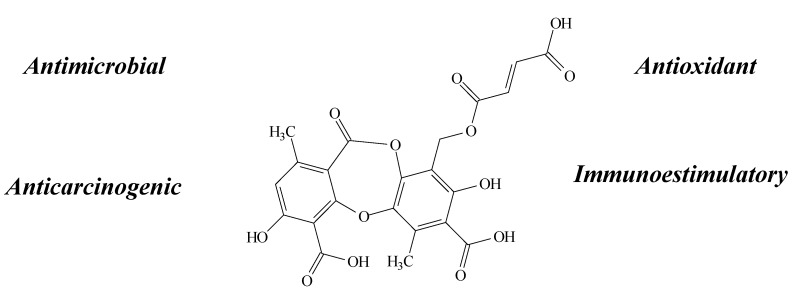
Fumarprotocetraric acid structure and biological activities.

Fumarprotocetraric acid (Figure 8) is a member of the depsidone family and can be isolated from *Cladonia verticillaris* Roddi, *C. rangiferina* and *Usnea articulata* [16,18,28].

Research about its properties revealed a stronger antioxidant (DPPH, SAS and reducing power assays) [16,28], antimicrobial (MIC assay) and anticarcinogenic (MTT assay) activity against the FemX and LS174 cell lines, consistent with the induction of apoptosis in a cell cycle-dependent manner [28]. In another study conducted by Santos *et al.* [18], furmaprotocetraric acid stimulated an increase of NO release in macrophage cells. Macrophages are known to play an important role in host defense mechanisms. In the immune system, reactive oxygen intermediates (ROI) often function together with nitric oxide (NO), for example in macrophage killing of bacteria and tumors cells, therefore inducing immunostimulatory effects [18].

### 2.8. Salazinic Acid

**Figure 9:**
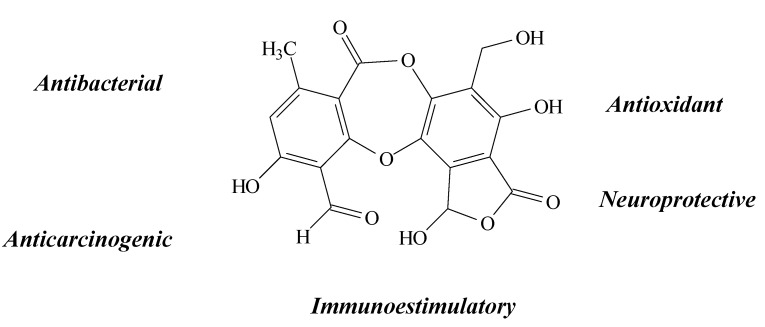
Salazinic acid structure and biological activities.

From the depsidone class, salazinic acid (Figure 9) can be isolated from *Xanthoparmelia camtschadalis*, *Rimelia cetrata* and *Parmelia caperata* [18,21,38]. This compound can be used as an antioxidant agent in Alzheimer’s disease for its benefits in decreasing ROS production in U373MG cells by hydrogen peroxide in the ORAC assay, inducing neuroprotection through their antioxidant ability in astrocytes and protecting against oxidative stress [21]. Santos *et al.* [18] also demonstrated that salazinic acid was able to activate the release of H_2_O_2_ and NO in a culture of mice peritoneal macrophages. These releases could involve the so-called oxidative burst (O_2_^−^), a sequence of biochemical reactions that ends with the production and extracellular release of ROS, playing an important role in macrophage killing of bacteria and tumors, and inducing immunostimulatory effects [18].

Furthermore, salazinic acid, in a study conducted by Manojlović *et al.* [38], demonstrated stronger antioxidant properties due to high activity on scavenging DPPH radicals, superoxide anion radical scavenging and reducing power, besides high antibacterial properties against *B. mycoides*, *B. subtilis*, *E. coli*, *K. pneumonia*, *S. aureus*, *A. flavus*, *A. fumigatus*, *C. albicans*, *P. purpurescens* and *P. verrucosum* (MIC assay). Cytotoxic activity was also verified against the Femx and LS174 cell lines in the MTT assay. The authors suggest that these activities could be due to its higher phenol content.

### 2.9. Physodic Acid

**Figure 10:**
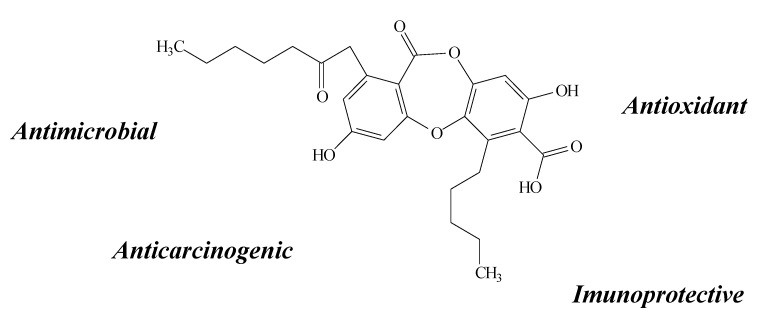
Physodic acid structure and biological activities.

Another depsidone compound, physodic acid (Figure 10) can be isolated from *Hypogymnia physodes*, and *Pseudoevernia furfuraceae* (L.) Zopf. Among its activities, antioxidant, imunoprotective, anticarcinogenic and antimicrobial properties can be observed [39,40,41].

Kosanić *et al.* [40] demonstrated high antioxidant activity in the reducing power and SAS assays, correlated with a high content of total phenolics of the acetone extracts of the species from which physodic acid was isolated. Likewise, very strong antimicrobial (MIC) assay activity against *B. mycoides*, *B. subtilis*, *E. coli*, *K. pneumoniae*, *S. aureus*, *A. flavus*, *A. fumigatus*, *C. albicans*, *P. purpurescens* and *P. verrucosum* was observed along with cytotoxic activities against human melanoma FemX and human colon carcinoma LS 174 cell lines (MTT assay and flow cytometry). However, no mechanism of action was clarified in this study. Stojanović *et al.* [41] also observed a certain anticarcinogenic activity through a significant decrease in the viability and proliferation of HeLa cells, which could be explained by the substitution of positions 1 and 6 in these compounds with long nonpolar substituents. Moreover, Pavlović *et al.* [39] verified that physodic acid decreased rat thymocytes proliferation, mediated by increased cytotoxicity that could be due to the increase of ROS levels and decrease of mithochondrial membrane potential (MMP), therefore, inducing immunoprotective effects.

### 2.10. Orcinol, Orsellinic Acid and other Orsellinates

**Figure 11:**
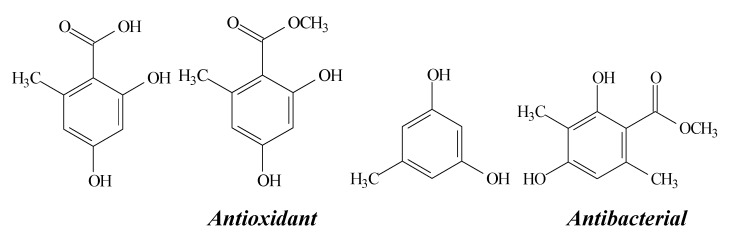
Structures and biological activities of orsellinic acid, methyl orsellinate, orcinol and methyl-β-orcinolcarboxylate, respectively.

Orsellinic acid and methyl orsellinate (Figure 11), were isolated from *Parmotrea stuppeum* by Jayaprakasha and Rao [31] and from *Heterodermia obscurata* by Thadhani *et al.* [7], together with methyl-β-orcinolcarboxylate and orcinol (benzoic acid derivatives and a benzenoid, respectively). Both studies observed antioxidant activity of the compounds in the β-carotene-linoleate model system [31] and in the NOR assay [7].

Orsellinate methyl-β-orsellinate or methyl-β-orcinolcarboxylate demonstrated antibacterial activity against *Staphilococcus aureus* in the MIC assay [24] and high activity in the NOR assay [7]. However, lower activity was demonstrated on the SOR assay and low or no effect was verified in the DPPH assay [27].

The relative inactivity in the SOR assay can be rationalized by observing the structures of the standards propyl gallate (PG) and butylated hydroxyanisole (BHA) which themselves possess only one aromatic ring. The latter two compounds, although mononuclear aromatic ones, have *ortho* and *para* OH/OR groups as compared to the *meta* relationship between OH/OR in the orsellinates, leading to higher SOR activity. On the other hand, mononuclear aromatic compounds were more active in the NOR assay suggesting that the redox potentials (LUMO) of simple aromatics are more compatible with the NO radical HOMO [7].

### 2.11. Other Compounds

Besides these major compounds, other less cited substances were also isolated from lichens and tested for antioxidant activity in the articles reviewed herein. We can classify all these compounds according to their antioxidant, antimicrobial and anticarcinogenic properties.

#### 2.11.1. Antioxidant

The depside evernic acid, isolated in research conducted by Kosanić *et al.* [40], demonstrated high antioxidant activity in reducing power and SAS assays, correlated with a high content of total phenolics of the acetone extracts of the species from which it was isolated. Other two depsides, sekikiac acid and erythrin, were isolated by Thadhani *et al.* [7], demonstrating good activity in SOR assay and NOR assays respectively.

Among the depsidones, norstistic acid, 8'-methylmenegazziaic, psoromic and protocetraric acid were the most active. Norstistic acid, isolated in the study of Dévéhat *et al.* [16] showed better SAS activity than quercetin. These results are in accordance with Ranković *et al.* [19] which also observed activity in the SAS and in the DPPH and reducing power assays. 8'-methylmenegazziaic exhibited higher antioxidant activity in the Co(II)/EDTA-induced luminol plateau chemiluminescence assay [29]. Meanwhile, psoromic acid showed antioxidant activity in the FRSA, NOR and LPO assays, and cardiovascular-protective effects in a concentration-dependent manner, in HMGR and ACE inhibition assays [17]. Protocetraric acid demonstrated higher activity in increasing NO release in macrophage cells, playing an important role in host defense mechanisms [18].

Kinoshita *et al.* [42] isolated four quinone derivatives from the lichens *Lethariella sernanderi*, *L. cashmeriana* and *L. sinensis*. Their antioxidant activity was assessed using a commercial assay kit based on Cu(II) reducing activity. Among them, 7-chlororubrocashmeriquinone showed the strongest potential, although canarione and 7-chlorocanarione also demonstrated high antioxidant activities, which suggested that the 1,2-quinone and 7-Cl moieties were important for antioxidant activity.

Ramalin (γ-glutamyl-*N*'-(2-hydroxyphenyl)hydrazide), a nitrogen compound, was isolated from the Antarctic lichen *Ramalina terebrata* [39]. According to Paudel *et al.* [43], ramalin presented scavenging activity against DPPH, 2,2'-azino-bis (3-ethylbenzthiazoline-6-sulfonic acid free radicals (ABTS•+), and superoxide anion radicals, and Fe^3+^ to Fe^2+^ ion reducing capacity. Furthermore, ramalin was able to inhibit the tyrosinase enzyme activity and showed no or very little cytotoxicity in human keratinocyte and fibroblast cells at its antioxidant concentration. These data suggest that ramalin had strong hydrogen- and electron-donating capacity, which may be the source of its very strong non-toxic antioxidant potential [43]. TRamalin could therefore be viewed as a potential product for future cosmetic and therapeutic applications.

From the xanthone class, Takenaka *et al.* [44] tested for DPPH free radical scavenging activity some compounds isolated from the lichen *Pyrenula japonica*. The activities of 1,5,8-trihydroxy-3-methylxanthone and 1,2,8-trihydroxy-5-methoxy-3-methylxanthone were higher than those of well-known antioxidants like α-tocopherol and 2,6-di(*tert*-butyl)-4-methylphenol (BHT), while 1,8-dihydroxy-5-methoxy-3-methylxanthone and 1,7-dihydroxy-3-methylxanthone showed low activities. The importance of the two hydroxyl groups in an *ortho*-diphenolic arrangement, which may be responsible for the antioxidant potential, is worth noting.

Marante *et al.* [24] used a phytotoxicity-based extraction and fractionation to separate allelochemicals contained in an extract of *Lethariella canariensis*. Among benzoic acid and its derivatives isolated, atranol, chloroatranol, methyl hematommate and ethyl hematommate exhibited a dose-dependent antioxidant activity in a LPO assay, protecting tissue against oxidative stress. Methyl haematommate also demonstrated NOR activity, along with another benzoic acid derivative, montagnetal, in the study of Thadhani *et al.* [7]. Given the large amounts of *p*-substituted polyphenolic compounds found in lichens and their antioxidant activity, it is conceivable that they contribute to the antioxidant defense mechanisms of these organisms [7].

#### 2.11.2. Antimicrobial

Among all substances, only the depside evernic acid and the depsidones protocetraric acid, nortistic acid and lobastin demonstrated some effects. Evernic, protocetraric and norstistic acid were active against the bacteria and fungi *B. mycoides*, *B. subtilis*, *E. coli*, *K. pneumonia*, *S. aureus*, *A. flavus*, *A. fumigatus*, *C. albicans*, *P. purpurescens*, *P. verrucosum* [19,39,41]. Meanwhile, lobastin was only active against the Gram-positive bacteria *Staphylococcus aureus* and *Bacillus subtilis* [37].

#### 2.11.3. Anticarcinogenic

From the depside class, Kosanić *et al.* [40] described evernic acid’s activity against FemX and LS174. Bačrová *et al.* [20] verified that gyrophoric acid was highly effective, cytotoxic and slightly pro-apoptotic against the HL-60, A2780 and Jurkat cell lines, which correlated with a cell cycle variation, represented by the accumation of cells in S-phase at the expense of the G_1_/G_0_-phase. Another depside, sphaerophorin, was able to trigger apoptotic death in melanoma cancer cells. In fact, a high DNA fragmentation (Comet and TUNEL Assays), reinforced by a significant increase in the caspase-3 enzyme activity, and not correlated to lactic dehydrogenase release, a marker of membrane breakdown, occurred in melanoma cells treated with these natural compound. Also, increased ROS formation was observed in a concentration-dependent manner, which could amplify the apoptosis cascades, besides a dose-dependent superoxide scavenging effect [45].

Also from the research of Russo *et al.* [45], the depsidone pannarin was isolated. Its activity was the same as that observed for sphaerophorin. These findings provide evidence that pannarin and sphaerophorin prevent UV light and nitric oxide-mediated plasmid DNA damage, and attenuate the growth of melanoma cells, at least in part, by triggering an apoptotic process.

**Table 1:** Characteristics of Included Studies.

Substance/Chemical Class	Authors, Year, Country	Source	Assay	Activity	Results/Mechanism of Action
Usnic acid (dibenzofuran)	Marante *et al.* [24], 2003, Spain	*Lethariella canariensis*	*In vitro*: MIC, MTT and LPO	Antibacterial, anticarcinogenic and antioxidant	Anti-proliferative effect against U937 and HL-60
	Santos *et al.* [18], 2004, Brazil	*Usnea meridionalis* Zahlbr	*In vitro*: H_2_O_2_ and NO measurements	Immunostimulatory	Induced greatest release of NO in peritoneal macrophages.
	Odabasoglu *et al.* [15], 2006, Turkey	*Usnea longissima*	*In vivo*: SOD, CAT, GR, GPx, MPx, NOS, GSH, and LPO	Gastroprotective	Increased SOD, GPx, GSH and cNOS activities and reduced CAT, GR, LPO, iNOS and MPx activities
	Dévéhat *et al.* [16], 2007, France	*Usnea articulata*	*In vitro*: DPPH and SAS	No significant activity	–
	Jin, Li and He [22], 2008, China	*Usnea longissma*	*In vitro*: MTT, TNF-α and NO	Anti-inflammatory	Dose-dependent inhibitory effect on LPS-induced TNF-α and NO production in macrophages RAW 264.7, associated with decreased synthesis of TNF-α mRNA and iNOS protein
	De Paz *et al.* [21], 2010, Spain	*Xanthoparmelia conspersa*	*In vitro*: MTT, ORAC and ROS determination	Antioxidant and neuroprotective	Reduced radical oxygen species (ROS) production on hydrogen peroxide-induced damage in U373 MG cells
	Bačkorová *et al*. [25], 2011, Slovakia	Purchased from Sigma Chemical	*In vitro*: MTT, HTCA, viability, cell proliferation and detachment, cell cycle transition and apoptotic nuclear morphology	Anticarcinogenic	Anti-proliferative action on A2780, MCF-7, SK-BR-3, HT-29, HCT-116 p53^−/−^, HL-60 and Jurkat. Higher pro-apoptotic activity, supported by the suppression of viability and cell proliferation, correlated more strongly with an increased number of floating cells
	Thadhani *et al.* [7], 2011, Sri-Lanka	*Parmotrema grayana*	*In vitro*: SOR, NOR, and DPPH	No significant activity	–
	Behera, Mahadik and Morey [17], 2012, India	*Usnea complanata*	*In vivo*: FRSA, NOR, LPO, ACE and HMGR	Cardioprotective	Moderate to strong antioxidant activity, concentration-dependent manner, on the FRSA, NOR) and in LPO. Poor fobrinolytic potencial
	Bessadottir *et al.* [20], 2012, Iceland	*Cladonia arbuscula*	*In vitro*: ATP estimation, immunocytochemistry, western blot, visualization of lysosomes and transfection with tfLC3 construct	Autophagy and pH-determined drug distribution	Induced the formation of autophagosomes in human cancer cells, but had minimal effects on normal human fibroblasts. UA-treated cells showed reduced ATP levels and activation of AMP kinase as well as signs of cellular stress. UA is thus likely to trigger autophagosome formation both by energy depletion and stress conditions.
	Brisdelli *et al.* [23], 2013, Italy	*Cladonia lepidophora* Ahti & Kashiw	*In vitro*: MTT, CAS 3, 8 and 9, ROS determination and DPPH	Anticarcinogenic	Anti-proliferative effect against MCF-7, HeLa, HCT-116
	Rabelo *et al.* [27], 2012, Brazil	Purchased from Sigma Chemical	*In vitro*: TRAP/TAR, OHRS, NOS, TBARS, SOD, CAT, MTT, DFCH-DA	Antioxidant and neurotoxicological	Induced cell detachment and loss of viability at higher concentrations (20 µg/mL) of SH-SY5Y cells alone or in the presence of H_2_O_2_ or 1% of FBS, related to the increase of intracellular ROS, inducing an oxidative stress scenario
	Polat *et al.* [26], 2013, Turkey	Purchased from Sigma Chemical	*In vitro*: TAC, TOS, CA and MN	Antioxidant	Increased TAC in low doses and TOS in a high dose
	Ranković *et al.* [19], 2012, Serbia	*Usnea barbata*	*In vitro*: DPPH, SAS, reducing power, MIC, MTT and flow cytometry	Antioxidant, antimicrobial and anticarcinogenic	Very strong antioxidant and antimicrobial activities. Antiproliferative activity correlated with an increase in the number of cells in the sub-G1 phase whiled the percentage of cells in the S-phase and G2/M phase remained unchanged compared to the controls. Interestingly, LS174 cells treated with the tested samples showed a significant increase of the sub-G1 phase and concomitant decrease in G2/M was observed, supporting a G1 phase arrest. These results suggested that the compound have a prominent ability to induce apoptosis in FemX and LS174 cells.
Atranorim(depside)	Marante *et al.* [24], 2003, Spain	*Lethariella canariensis (Parmeliaceae)*	*In vitro*: MIC, MTT and LPO	Antibacterial, anticarcinogenic and antioxidant	Moderate antibacterial activity against *Staphylococcus aureus.* Anti-proliferative effect against U937 and HL-60 and a dose-dependent antioxidant activity (100–250 µM) by decreasing H_2_O_2_/FeCl_2_ and inhibiting LPO, protecting from oxidative damage by the inhibition of both ROS and free radicals
	Dévéhat *et al.* [16], 2007, France	*Usnea articulata;*	*In vitro*: DPPH and SAS	No significant activity	–
	Papadopoulou *et al.* [29], 2007, Greece	*Hypotrachyna revoluta*	*In vitro*: CO(II)/EDTA induced luminol hemiluminescence	Antioxidant	Antioxidant effect due to an additional hydroxyl group on the aromatic ring were the most active ones
	Bačkorová *et al.* [25], 2011, Slovakia	Purchased from Sigma Chemical Co.	*In vitro*: MTT, HTCA, viability, cell proliferation and detachment, cell cycle transition and apoptotic nuclear morphology	Anticarcinogenic	Evoked cytotoxicity in HL-60, A2780, MCF-7, SK-BR-3, HT-29, HCT-116 p53^−/−^, HCT-116 p53^+/+^ and Jurkat, triggered by higher pro-apoptotic activity (except on A2780 cell line), and supported by the inhibition of clonogenic ability and cell proliferation
	Thadhani *et al.* [7], 2011, Sri-Lanka	*Parmotrema grayana*	*In vitro*: SOR, NOR, and DPPH	No significant activity	–
	Melo *et al.* [8], 2011, Brazil	*Cladina kalbii*	*In vitro*: TRAP/TAR, TBARS, HRS, NOS, CAT, SOD, MTT, and LPO	Antioxidant, cytoprotective and pro-oxidative	Superoxide dismutase-like and scavenging activity of peroxyl radicals. Induce cytoprotection in the presence of toxic concentrations of H_2_O_2_ on the SH-SY5Y cells. Conversely, it presented a pro-oxidant capacity in a lipid-rich system, enhancing TBARS and also enhanced production of NO and H_2_O_2_ in higher concentrations
	Barreto *et al.* [30], 2013, Brazil	*Cadina Kalbi*	*In vivo*: Myofibroblast field and macroscopic and histological analyses	Wound healing	Topical application of atranorin reduced wound areas, induced earlier granulation tissue formation, increased cell proliferation, improved collagenization and modulated the myofi broblasts differentiation when compared to control animals.
	Kosanić *et al.* [28], 2014, Serbia	*Cladonia furcata*	*In vitro*: DPPH, SAS, reducing power, MIC, MTT and flow cytometry	Antioxidant, antimicrobial and anticarcinogenic	Very strong antioxidant and antimicrobial activities. Antiproliferative activity accompanied by a stronger increase in the percentage of the sub-G1 population and concomitant decrease in G2/M of FemX and LS174 cell lines, leading to a G0/G1 cell cycle block and inducing apoptosis in a cell cycle-dependent manner
Diffractaic acid (depside)	Santos *et al.* [18], 2004, Brazil	*Usnea subvacata* Motyka	*In vitro*: H2O2 and NO measurements	Immunostimulatory	Induced greatest release of NO in peritoneal macrophages
	Bayir *et al.* [33], 2006, Turkey	*Usnea longissima*	*In vivo*: SOD, GPx, GSH, LPO, MPx, NOS, iNOS cNOSand CAT	Antioxidant and gastroprotective	Decreased MPx, iNOS and LPO and increased cNOS, SOD, GPx and GSH, inhibiting neutrophil infiltration into gastric mucosal tissues
	Odabasoglu *et al.* [36], 2012, Turkey	*Usnea longissima*	*In vivo*: TUNEL, GSH, SOD, iNOS, MPx, CAS 2, CAS 3, CAS 8, and CAS 9	Anticarcinogenic	Reduced the iNOS and MPx activities and increased SOD, GSH level and caspases (2, 3, 8 and 9) activities in tissue surrounding titanium implants
	Brisdelli *et al.* [23], 2013, Italy	*Protousnea magellanica* (Mont.) Krog	*In vitro*: MTT, CAS 3, 8 and 9, ROS determination and DPPH	Anticarcinogenic	Antiproliferative activity against HCT-116 cells and reduction of viability in MCF-7 and HeLa cells
Lecanoric acid (depside)	Santos *et al.* [18], 2004, Brazil	*Usnea subvacata* Motyka	*In vitro*: H_2_O_2_ and NO measurements	No significant activity	–
	Jayaprakasha and Rao [31], 2000, India	*Parmotrea stuppeum*	*In vitro*: β-carotene/linoleate model	Antioxidant	Moderate antioxidant activity
	Thadhani *et al.* [7], 2011, Sri-Lanka	*Parmotrema grayana*	*In vitro*: SOR, NOR, and DPPH	Antioxidant	Presented a high SOR activity, comparable to the standards of Propyl gallate (PG) and Butyrated hydroxyanisole (BHA). And moderate activity on NOR and DPPH.
	Lopes *et al.* [32], 2008, Brazil	*Parmotrema tinctorum*	*In vitro*: DPPH	Antioxidant	Moderate activity with an IC_50_ of 42.87 ± 1.20
Stictic acid (depsidone)	De Paz *et al.* [21], 2010, Spain	Xanthoparmelia camtschadalis	*In vitro*: MTT, ORAC and ROS determination	Antioxidant and neuroprotective	Protective effect against U373MG cell line by decreasing ROS production induced by H_2_O_2_
	Papadopoulou *et al.* [29], 2007, Greece	*Hypotrachyna revoluta*	*In vitro*: CO(II)/EDTA induced luminol chemiluminescence	Antioxidant	Noteworthy antioxidant activity
	Dévéhat *et al.* [16], 2007, France	*Usnea articulata*	*In vitro*: DPPH and SAS	No significant activity	–
Lobaric acid (depsidones)	Bhattarai *et al.* [37], 2013. Republic of Korea	*Stereocaulon alpinum*	*In vitro*: Paper disk diffusion, MTT and DPPH	Antibacterial and antioxidant	Activity against gram-positive bacteria *Staphylococcus aureus* and *Bacillus subtilis* and moderate scavenge activity in a dose-dependent manner without any toxic effects
	Brisdelli *et al.* [23], 2013, Italy	*Stereocaulon alpinum* Laurer ex Funck	*In vitro*: MTT, CAS 3, 8 and 9, ROS determination and DPPH	Anticarcinogenic	Anti-proliferative activity against HeLa and HCT cells lines
	Thadhani *et al.* [7], 2011, Sri-Lanka	*Cladonia sp.*	*In vitro*: SOR, NOR, and DPPH	Antioxidant	Promising antioxidant activity in SOR assay
Methyl orsenillate (Benzenoid)	Jayaprakasha and Rao [31], 2000, India	*Parmotrea stuppeum*	*In vitro*: β-carotene/linoleate model	Antioxidant	Moderate antioxidant activity
Orsenillic acid (Benzoic acid derivative)					
Orcinol (1) (Benzenoid)	Thadhani *et al.* [7], 2011, Sri-Lanka	*Parmotrema grayana*	*In vitro*: SOR, NOR, and DPPH	Antioxidant	Activity in NOR assay
Orsellinic acid (2) (Benzenoid)		*Parmotrema grayana,*			Activity in NOR assay
Methyl orsellinate (3) (Benzoic acid derivative)		*Heterodermia obscurata*			Activity in NOR assay
Methyl haematommate (4) Benzoic acid derivative)		*Heterodermia obscurata*			Activity in NOR assay
Methyl β-orcinolcarboxylate (5) (Benzoic acid derivative)		*Heterodermia obscurata*			Activity in NOR assay
Methyl β-orsellinate (Benzoic acid derivative)	Marante *et al.* [24], 2003, Spain	*Lethariella canariensis*	*In vitro*: MIC, MTT and LPO	Antibacterial	Activity against *Staphilococcus aureus*
Protocetraric acid (depsidone)	Manojlović *et al.* [38], 2012, Serbia	*Parmelia caperata*	*In vitro*: DPPH, reducing power, SAS, MIC and MTT	Antibacterial and anticarcinogenic	Highly antibacterial active and presented strong anticancer activity toward FemX and LS174 cell lines. These activities could be due to its higher phenol content.
	Santos *et al.* [18], 2004, Brazil	*Usnea subvacata* Motyka	*In vitro*: H_2_O_2_ and NO measurements	Immunostimulatory	Higher activity in increasing NO release in macrophage cells.
Fumarprotocetraric acid (depsidone)		*Cladonia verticillaris* Roddi			Higher activity in increasing NO and H_2_O_2_ release in macrophage cells
	Dévéhat *et al.* [16], 2007, France	*Usnea articulata*	*In vitro*: DPPH and SAS	Antioxidant	High activity in the SAS and moderate in the DPPH
	Kosanić *et al.* [28], 2014, Serbia	*Cladonia rangiferina*	*In vitro*: DPPH, SAS, reducing power, MIC, MTT and flow cytometry	Antioxidant, antimicrobial and anticarcinogenic	Strong antioxidant and antimicrobial activities. Antiproliferative activity accompanied by a stronger increase in the percentage of the sub-G1 population and concomitant decrease in G2/M of FemX and LS174 cell lines, inducing apoptosis in a cell cycle-dependent manner
Cryptostictinolide (Compound 2-C_19_H_16_O_8_ - depsidone)	Dévéhat *et al.* [16], 2007, Australia			Antioxidant	Moderate activity in DPPH
Compound 1 (C_19_H_14_O_8_, identical to stictic acid–depsidone)				No significant activity	Moderate activity in DPPH
Cryptostictic acid (Depsidone)				No significant activity	–
Menegazziaic acid (Depsidone)				No significant activity	–
Constictic acid (Depsidone)				No significant activity	–
3-*O*-methylconsalazinic acid (Depsidone)				No significant activity	–
Barbatic acid (Depside)				No significant activity	–
Ergosterol peroxide (Terpenoid)				No significant activity	–
Peristictic acid (Depsidone)				Antioxidant	–
Norstictic acid (depsidone)				Antioxidant	High SAS activity
	Ranković *et al.* [19], 2012, Serbia	*Toninia candida*	*In vitro*: DPPH, SAS, reducing power, MIC, MTT and flow cytometry	Antioxidant, antimicrobial and anticarcinogenic	Stronger antioxidant and antimicrobial activities. Antiproliferative activity correlated with an increase in the number of cells in the sub-G1 phase whiled the percentage of cells in the S-phase and G2/M phase remained unchanged compared to the controls. Interestingly, LS174 cells treated with the tested samples showed a significant increase of the sub-G1 phase and concomitant decrease in G2/M was observed, supporting a G1 phase arrest. These results suggested that the compound have a prominent ability to induce apoptosis in FemX and LS174 cells.
Cryptostictinolide (depsidone)	Papadopoulou *et al.* [29], 2007, Greece	*Hypotrachyna revoluta* (Flörke) Hale	*In vitro*: CO(II)/EDTA induced luminol chemiluminescence	No significant activity	–
Hypotrachynic acid (depside)				Antioxidant	Noteworthy antioxidant activity
Deoxystictic acid (depsidone)				Antioxidant	Noteworthy antioxidant activity
8'-methylconstictic acid (depsidone)				Antioxidant	Noteworthy antioxidant activity
8'-methylstictic acid (depsidone)				Antioxidant	Noteworthy antioxidant activity
8'-methylmenegazziaic acid (depsidone)				Antioxidant	Noteworthy antioxidant activity
8'-ethylstictic acid (depsidone)				Antioxidant	Noteworthy antioxidant activity
Atranol (benzoic acid derivative)	Marante *et al.* [24], 2003, Spain	*Lethariella canariensis*	*In vitro*: MIC, MTT and LPO	Anticarcinogenic	Inhibited the proliferation of U937 and HL-60 and presented dose-dependent antioxidant activity
Chloroatranol (benzoic acid derivative)				Anticarcinogenic and antioxidant	Inhibited the proliferation of U937 and HL-60 and presented dose-dependent antioxidant activity
Hematommic acid (benzoic acid derivative)				Antioxidant	Dose-dependent antioxidant activity
Chlorohematommic acid (benzoic acid derivative)				Antioxidant	Dose-dependent antioxidant activity
Methyl chlorohematommate (benzoic acid derivative)				Antioxidant	Dose-dependent antioxidant activity
Ethyl hematommate (benzoic acid derivative)				Anticarcinogenic and antioxidant	Inhibited the proliferation of U937 and HL-60 and presented dose-dependent antioxidant activity
Ethyl chlorohematommate (benzoic acid derivative)				Antioxidant	Dose-dependent antioxidant activity
Chloroatranorin (benzoic acid derivative)				Antioxidant	Dose-dependent antioxidant activity
Methyl hematommate (benzoic acid derivative)				Anticarcinogenic and antioxidant	Inhibited the proliferation of U937 and HL-60 and presented dose-dependent antioxidant activity
	Thadhani *et al.* [7], 2011, Sri-Lanka	*Cladonia sp.*	*In vitro*: SOR, NOR, and DPPH	Antioxidant	Promising antioxidant activity in NOR
Montagnetal (benzoic acid derivative)				Antioxidant	Promising antioxidant activity in NOR
Divericatic acid (depside)				Antioxidant	Significant level of activity in SOR
Erythrin (depside)				Antioxidant	Promising antioxidant activity in NOR
Sekikiac acid (depside)				Antioxidant	Significant level of activity in SOR
Zeorin (Terpenoid)				Antioxidant	Significant level of activity in SOR
Lobastin (depsidone)	Bhattarai *et al.* [37], 2013. Republic of Korea	*Stereocaulon alpinum*	*In vitro*: Paper disk diffusion, MTT and DPPH	Antibacterial and Antioxidant	Active against Gram-positive bacteria, B. subtilis and S. aureus. Moderate antioxidant activity compared with the synthetic commercial standard BHT
Sphaerophorin (depside)	Russo *et al.* [44], 2008, Chile	*Sphaerophorus globosus, Psoroma reticulatum, P. pulchrum, P. balladium*	*In vitro*: DNA cleavage induced by H_2_O_2_ UV-photolysis, DNA single-strand breaks induced by Angeli’s salt, SAS, TUNEL, Comet, CAS 3, ROS determination; SAS, MTT	Antioxidant and Anticarcinogenic	The compounds suppressed the formation of lin DNA and induced a partial recovery of scDNA; showed a dose-dependent superoxide scavenging effect; exhibited a significant inhibitory effect on M14 cell; produced DNA damage, inducing a programmed cell death; increased cas-3 and ROS in a concentrantion-dependent manner.
Pannarin (depsidone)					
Psoromic acid (depsidone)	Behera, Mahadik and Morey [17], 2012, India	*Usnea complanata*	*In vivo*: FRSA, NOR, LPO, ACE and HMGR	Cardioprotective	Moderate to strong antioxidant activity, concentration-dependent manner, on the FRSA, NOR and in LPO. Poor fibrinolytic potential
Isophysodic acid (depsidone)	Pavlović *et al.* [39], 2013, Serbia	*Hypogymnia physodes*	*In vitro*: CCK-8, ROS determination, MMP	No significant activity	–
Physodalic acid (depsidone)				Immunoprotective	Induced thymocytes toxicity mainly through increased ROS levels and decreased MMP
	Stojanović *et al.* [41], 2014, Serbia		*In vitro*: MTT	No significant activity	–
3-hydroxyphysodic acid (depsidone)	Pavlović *et al.* [39], 2013, Serbia		*In vitro*: CCK-8, ROS determination, MMP	Immunoprotective	Induced thymocytes toxicity that may lead to intracellular low energy levels with resulted cytotoxicity
	Stojanović *et al.* [41], 2014, Serbia		*In vitro*: MTT	Anticarcinogenic	Anti-proliferative action on HeLa cells
Physodic acid (depsidone)	Pavlović *et al.* [39], 2013, Serbia		*In vitro*: CCK-8, ROS determination, MMP	Immunoprotective	Induced thymocytes toxicity mainly through increased ROS levels and decreased MMP
	Stojanović *et al.* [41], 2014, Serbia		*In vitro*: MTT	Anticarcinogenic	Anti-proliferative action on HeLa cells
	Kosanić *et al.* [40], 2013, Serbia	*Pseudoevernia furfuraceae* (L.) Zopf	*In vitro*: DPPH, Reducing power, SAS, MIC, MTT and flow citometry	Antioxidant, antimicrobial and anticarcinogenic	Both compounds showed high antioxidant activity on reducing power and SAS assays, correlated with a high content of total phenol of the acetone extracts of the species from which they were isolated. Very strong antimicrobial (MIC) assay activity against B. mycoides, B. subtilis, *E. coli*, K. neumonia, S. aureus, A. flavus, A. fumigatus, C. albicans, P. purpurescens and P. verrucosum, and cytotoxic activities against FemX and LS 174 cell lines.	
Evernic acid (depside)					
Parietin (quinone)	Bačkorová *et al.* [25], 2011,Slovakia	*Xanthoria parietina*	*In vitro*: MTT, HTCA, viability, cell proliferation and detachment, cell cycle transition and apoptotic nuclear morphology	Anticarcinogenic	Evoked cytotoxicity in A2780, Jurkat and HT-29 human cancer cell lines. Only inhibited some clonogenic ability of HeLa and MCF-7 cell lines.
Gyrophoric acid (depside)		*Umbilicaria hirsuta*		Anticarcinogenic	Anti-proliferative effect on HL-60, A2780 and Jurkat cells and and slightly pro-apoptotic.
Vicanicin (depsidones)	Brisdelli *et al.* [23], 2013, Italy	*Psoroma pallidum* Nyl., *P. pulchrum Malme*	*In vitro*: MTT, CAS 3, 8 and 9, ROS determination and DPPH	Anticarcinogenic	Induced a significant loss of viability in a dose-dependent manner in HeLa and HCT-116 cells
Variolaric acid		*Ochrolechia deceptionis* (Hue) Darb.		No significant activity	–
Protolichesterinic acid (depsidones)		*Cornicularia aculeata* (Schreb.) Ach.		Anticarcinogenic	Stronger cytotoxic activity related to its ability to induce apoptosis in HeLa cells by activating an extrinsic cas-8/-3-mediated as well as intrinsic cas-9/-3-mediated pathway.
Salazinic acid (depsidone)	De Paz *et al.* [21], 2010, Spain	*Xanthoparmelia camtschadalis*	*In vitro*: MTT, ORAC and ROS determination	Antioxidant and neuroprotective	Protective effect against U373MG cell line by decreasing ROS production induced by H_2_O_2_
	Manojlović *et al.* [38], 2012, Serbia	*Parmelia caperata*	*In vitro*: DPPH, reducing power, SAS, MIC and MTT	Antibacterial and anticarcinogenic	Active against B. mycoides, B. subtilis, *E. coli*, K. neumonia, S. aureus, A. flavus, A. fumigatus, C. albicans, P. purpurescens and P. verrucosum and presented anti-proliferative activity toward FemX and LS174 cell lines.
	Santos *et al.* [18], 2004, Brazil	*Rimelia cetrata*	*In vitro*: H_2_O_2_ and NO measurements	Immunostimulatory	Activated the release of H_2_O_2_ and NO in the culture of mice peritoneal macrophages.
Hypostictic acid (depsidone)		*Pseudoparmelia sphaerosphora* (Nyl) Hale		No significant activity	–
Biruloquinone (quinone)	Luo *et al.* [46], 2013, China	*Cladonia macilenta*	*In vitro*: MTT and AChE inhibittory	Neuroprotective	Improved viability the H_2_O_2_ and β-amyloid injured PC12 cells. Classified as a a mixed-II inhibitor
Canarione (quinone)	Kinoshita *et al.* [42], 2010, Japan	*Lethariella sernanderi, L. cashmeriana, and L. sinensis*	*In vitro*: Cu(II) reducing activity	Antioxidant	Among them, 7-chlororubrocashmeriquinone showed the strongest potential, although canarione, 7-chlorocanarione also demonstrated high antioxidant activities
Rubrocashmeriquinone (quinone)					
7-Chlororubrocashmeriquinone (quinone)					
7-chlorocanarione (quinone)					
Ramalin (nitrogen compound)	Paudel *et al.* [43], 2011, Republic of Korea	*Ramalina terebrata*	*In vitro*: DPPH, ABTS^•+^, Fe^3+^ reducing power, SAS, and tyrosinase inhibitory *In vivo*: MTT, H_2_O_2_ and iNOS	Antioxidant and anticarcinogenic	Scavenged DPPH, ABTS^•+^, NO and H_2_O_2_ radicals. Presented capacity in reducing Fe^3+^ to Fe^2+^ ions and inhibited tyrosinase activity in murine macrophage
1,8-dihydrixy-3-hydroxymethyl-5-methylxanthone (xantone)	Takenaka *et al.* [44], 2000, Japan	*Pyrenula japonica*	Not tested	–	–
1,2,8-trihydroxy-5-methoxy-3-methylxanthone (xantone)			*In vitro*: DPPH	Antioxidant	Higher scavenging than those well-known antioxidants, α-tocopherol and BHT
1,7-dihydroxy-3-methylxanthone (xantone)			*In vitro*: DPPH	–	Low scavenging activity
1,5,8-trihydroxy-3-methylxanthone (xantone)			*In vitro*: DPPH	Antioxidant	Higher scavenging than those well-known antioxidants, α-tocopherol and BHT
1,8-dihydroxy-5-methoxy-3-methylxanthone (xantone)			*In vitro*: DPPH	–	Low scavenging activity
Emodin (xantone)			Not tested	–	–
Sclerotiorin (xantone)			Not tested	–	–

Definition of abbreviations: A2780 = ovarian carcinoma, ABTS^•+^ = 2,2'-azino-bis (3-ethylbenzthiazoline-6-sulfonic acid free radicals, BHA = Butyrated hydroxyanisole, cNOS = constitutive nitric oxide synthase, BHT = 2,6-di(terc-butyl)-4-methylphenol, CAS = caspase, CAT = catalase assay, CCK8 = *Cell Counting Kit-8*, DCFH-DA = 2',7'-dichlorofluorescein diacetate assay, DNA = deoxyribonucleic acid, DPPH = 2,2-diphenyl-1-picrylhydrazil radical scavenging, Fe+^2^ = ferrous ion, FemX = human melanoma, FRSA = Free Radical Scavenge Activity, GPx = glutathione peroxidase assay, GR = Glutatione reductase activity, GSH = glutathione assay, H_2_O_2_ = hydrogen peroxide, HCT = colon carcinoma, HCT-116 = colon carcinoma, HCT-116 p53^−/−^ = human colon carcinoma p53-null subline, HeLa = cervix adenocarcinoma, HeLa = human cervix adenocarcinoma, HL-60 = human monocytic leukemia, HMGR = Hydroxy-3-methyl-glutaryl-CoA reductase, HRS = hydroxyl radical-scavenging activity, HT-29 = human colon adenocarcinoma, HTCA = human tumor clonogenic assay, iNOS = Inducible nitric oxide synthase, Jurkat = human T cells lymphocyte leukaemia, LPO = lipid peroxidation assay, LS174 = human colon carcinoma, M14 = melanoma, MCF-7 = breast adenocarcinoma, MIC = Minimum inhibitory concentration assay, MMP = mitochondrial membrane potential assay, MPx = Myeloperoxidase, MTT = [3-(4,5-dimethylthiazol-2-yl)-2,5-diphenyltetrazolium bromide], NO = nitric oxide, NOR = nitric oxide radical scavenging, ORAC = Oxygen Radical Absorbance Capacity, PC12 = Rat adrenal phaeochromocytoma, PG = Propyl gallate, ROS = reactive oxygen species, SAS = Superoxide anion scavenging assay, *SH-SY5Y* = neuroblastoma, *SK-BR-3* = Human breast adenocarcinoma, SOD = superoxide dismutase assay, SOR = superoxide radical scavenging, TAC = Total antioxidant capacity, TAC = Total antioxidant capacity, TAR = total antioxidant reactivity index, TBARS = thiobarbituric acid reactive species, TNF-α = tumor necrosis factor-α, TOS = total oxidative status, TRAP = total reactive antioxidant potential index, TUNEL = terminal deoxynucleotidyl transferase (TdT)-mediated dUTP nick-end-labeling, U373MG = Human glioblastoma astrocytoma, U937 = human monocytic leukemia.

Other compounds from the depsidone family were also mentioned. Protocetraric acid demonstrated cytotoxicity against the FemX and LS174 cell lines [38]. Norstictic acid also presented antiproliferative activity against FemX and LS174 which may occur through an apoptosis pathway [18]. Physodalic acid significantly decreased rat thymocyte proliferation, mediated by increased cytotoxicity that could be due to the increase of ROS levels and decrease of mithochondrial membrane potential (MMP) [39]. 3-Hydroxyphysodic acid also decreased rat thymocyte proliferation and HeLa cell lines, however, it is speculated that its application to cell culture may not induce ROS generation, crucial for cell death, but at certain concentrations may lead to low intracellular energy levels with resulting cytotoxicity [39,41]. Vicanicin induced a significant loss of viability in a dose-dependent manner in HeLa and HCT-116 cells and protolichesterinic acid induced apoptosis in HeLa cells by activating an extrinsic caspases-8/-3-mediated as well as intrinsic caspases-9/-3-mediated pathway [23].

Benzoic acid and its derivatives atranol, chloroatranol, hematommic acid, chlorohematommic acid, methyl hematommate, methyl chlorohematommate, ethyl hematommate, ethyl chlorohematommate and chloroatranorin isolated in the study of Marante *et al.* [24] inhibited the proliferation of U937 and HL-60 monocytic leukemia cell lines.

Concerning the quinones, in the study of Bačrová *et al.* [25], parietin demonstrated significant cytotoxicity in the A2780, Jurkat and HT-29 cell lines. Although it did affect cell proliferation, the impact on cell viability and percentage of floating cells was insignificant. Therefore, the action of parietin may best be regarded as cytostatic. Biruloquinone, an *ortho*-phenanthraquinone compound was isolated by Luo *et al.* [46]. The AChE inhibitory assay classified this compound as a mixed-II inhibitor. An MTT assay verified that biruloquinone improved the viability of the H_2_O_2_ and β-amyloid injured PC12 cells. Due to its potent antioxidant activity, the authors suggested that biruloquinone may be used in the treatment of Alzheimer’s disease patients for enhancing their cognition and slowing the symptoms by protecting the injured neurons.

## 3. Final Considerations

Within the 32 studies here reviewed, eight classes of secondary metabolites were described: dibenzofurans, represented by usnic acid; depsides, represented by 13 different compounds; depsidones, by 28 compounds; benzoic acid derivatives, by 20 compounds; xanthones, by seven compounds; quinones, by six compounds; terpenoids, by two compounds; and nitrogen compound, represented by ramalin. The most cited substance was the usnic acid, mentioned in 14 studies, followed by atranorin, described in eight.

The pharmacological and other biological activities of lichen substances in this review could be divided into the following categories: antimicrobial activity, anticarcinogenic and antioxidant, which includes all the enzyme inhibitory activities involved in cytoprotective, cardioprotective, gastroprotective, and immunostimulatory properties. The wide variety of biological activities of lichens is generally correlated to their special ecological circumstances. Geographic, altitudinal or microhabitat conditions and even the presence or absence of light can cause differentiations in lichen physiology and metabolism. The phenolic content and distribution is also modified according to direct UV irradiation [47,48]. Consequently, the response of each isolated compound in the enormous variety of specific tests can differ among the different lichen species or related groups.

For the antioxidant assays it was observed that the DPPH, SAS, SOR and NOR tests were the most common ones used in the articles discussed here. However, other major assays were also used such as the Co(II)/EDTA-induced luminol plateau chemiluminescence assay, ORAC assay, TRAP/TAR, OHRS, TBARS, DFCH-DA, TAC/TOS, β-carotene/linoleate model, HMGR and AChE inhibition, reducing power, total phenolic content, ROS determination, H_2_O_2_ and NO measurements, CCK-8, ABTS^•+^ and the determination of some enzymes as SOD, CAT, GR, GPx, MPx, NOS, cNOS, iNOS, LPO and GSH. Most antibacterial activity was assessed by the MIC assay, apart from the paper disk infusion assay described in the study of Bhattarai *et al.* [37]. As for the anticarcinogenic tests, MTT was the most described cytotoxicity assay, used in almost all the studies discussed herein, except for those of Odabasoglu *et al.* [36] and Russo *et al.* [46] in which TUNEL and Comet assays were utilized instead. However, other tests were assessed in a way to describe the compound’s mechanism of action, such as HTCA, cell viability, cell proliferation, cell cycle transition, flow cytometry and caspases activities.

Although several studies utilized the same assays for the same compounds, there were discrepancies among the results observed, more specifically for the antioxidant assays. One possible explanation would concern the IC_50_ values. The several studies presented different standard or control compounds and also divergent IC_50_ values considered statistically significant. Thus, contrasting responses should be expected. For the reason, the activity of a compound must always be assessed with different tests, in order to identify different mechanisms and to establish in which degree a given compound interacts with the different reactive species.

Concerning the mechanisms of action by which these lichen compounds exert such activities, most of them are still not clear. However, it seems important that the higher phenol content in these compounds could be responsible for its antioxidant properties. Also, the number and position of OH groups in the aromatic ring(s) of the compounds is a common feature that may also account for some of their activities. Most of the lichenic compounds currently studied present a polyphenolic structure combined with acidic groups capable of exchanging protons with the environment.

## 4. Methods

This systematic review was conducted in accordance with the guidelines of Transparent Reporting of Systematic Reviews and Meta-Analyses (PRISMA statement) [49].

### 4.1. Search Strategy

Three internet sources were used to search for appropriate papers that met the study purpose. These included the National Library of Medicine, Washington, D.C. (MEDLINE-PubMed), Web of Science and LILACS (Latin American and Caribbean Health Sciences), using different combinations of the following keywords in medical subjects heading (MeSH) included: “Lichens”, “Antioxidant Response Elements”, and “Antioxidants”. The databases were searched for studies conducted in the period up to and including February, 2014. The structured search strategy was designed to include any published paper that evaluated the use of natural compounds obtained from lichens in antioxidant activity to identify those that show therapeutic potential. We did not contact investigators and did not attempt to identify unpublished data.

### 4.2. Study Selection

All electronic search titles, selected abstracts and full-text articles were independently reviewed by a minimum of two reviewers (J.S.S.Q. and M.R.V.S.). Disagreements on study inclusion/exclusion were resolved with a consensus reaching. The following inclusion criteria were applied: antioxidant activity studies and the use of natural compounds obtained from Lichens. Studies were excluded according to the following exclusion criteria: studies in humans, studies using extracts, mixtures of lichen compounds, synthetic or derived compounds, review articles, meta-analyses, abstracts, conference proceedings, editorials/letters and case reports (Figure 1).

### 4.3. Data Extraction

Data were extracted by one reviewer using standardized forms and were checked for completeness and accuracy by a second reviewer. Extracted information included data regarding the substance, source (lichen species), chemical classes of secondary metabolites, assay type, pharmacological activity studied, and effect and/or mechanism of action suggested.

## 5. Conclusions

Lichens are clearly an interesting and rich source of compounds; their properties clearly indicate their potential for pharmaceutical purposes, although some activities of lichens still need consideration. Further studies are still needed to clarify the molecular processes and signaling pathways involved in their activity. Thus, new compounds will certainly be described from poorly studied lichens, and even in species that are commonly used and chemically well known, new chemical components are still being detected.

## Supplementary Mterials

Supplementary materials can be accessed at: http://www.mdpi.com/1420-3049/19/9/14496/s1.

## Supplementary Files

Supplementary File 1

## References

[1] McCord J.M. (2000). The evolution of free radicals and oxidative stress. Am. J. Med..

[2] Alam M.N., Bristi N.J., Rafiquzzaman M. (2013). Review on *in vivo* and *in vitro* methods evaluation of antioxidant activity. Saudi Pharm. J..

[3] Lotito S.B., Frei B. (2006). Consumption of flavonoid-rich foods and increased plasma antioxidant capacity in humans: Cause, consequence, or epiphenomenon?. Free Radic. Biol. Med..

[4] Lobo V., Patil A., Phatak A., Chandra N. (2010). Free radicals, antioxidants and functional foods: Impact on human health. Pharmacogn. Rev..

[5] Brewer M.S. (2011). Natural Antioxidants: Sources, Compounds, Mechanisms of Action, and Potential Applications. Compr. Rev. Food Sci. Food Saf..

[6] Hale L.J. (1973). The pattern of growth of *Clytia johnstoni*. J. Embryol. Exp. Morphol..

[7] Thadhani V.M., Choudhary M.I., Ali S., Omar I., Siddique H., Karunaratne V. (2011). Antioxidant activity of some lichen metabolites. Nat. Prod. Res..

[8] Melo M.G.D., Santos J.P.A., Serafini M.R., Caregnato F.F., Pasquali M.A.B., Rabelo T.K., Rocha R.F., Quintans-Junior L.J., Araújo A.A.S., Silva F.A. (2011). Redox properties and cytoprotective actions of atranorin, a lichen secondary metabolite. Toxicol. In Vitro.

[9] Molnár K., Farkas E. (2010). Current results on biological activities of lichen secondary metabolites: A review. Z. Naturforsch. C.

[10] Schmitt I., Lumbsch H.T. (2004). Molecular phylogeny of the Pertusariaceae supports secondary chemistry as an important systematic character set in lichen-forming ascomycetes. Mol. Phylogenet. Evol..

[11] Shukla V., Joshi G.P., Rawat M.S.M. (2010). Lichens as a potential natural source of bioactive compounds: A review. Phytochem. Rev..

[12] Nunes P.S., Albuquerque-Junior R.L., Cavalcante D.R., Dantas M.D., Cardoso J.C., Bezerra M.S., Souza J.C., Serafini M.R., Quitans-Junior L.J., Bonjardim L.R. (2011). Collagen-based films containing liposome-loaded usnic acid as dressing for dermal burn healing. J. Biomed. Biotechnol..

[13] Nunes P.S., Bezerra M.S., Costa L.P., Cardoso J.C., Albuquerque-Junior R.L., Rodrigues M.O., Barin G.B., Silva F.A., Araujo A.A.S. (2010). Thermal characterization of usnic acid/collagen-based films. J. Therm. Anal. Calorim..

[14] Barreto R.S.S., Albuquerque-Júnior R.L.C., Araújo A.A.S., Almeida J.R.G.S., Santos M.R.V., Barreto A.S., DeSantana J.M., Siqueira-Lima P.S., Quintans J.S.S., Quintans-Júnior L.J. (2014). A Systematic Review of the Wound-Healing Effects of Monoterpenes and Iridoid Derivatives. Molecules.

[15] Odabasoglu F., Cakir A., Suleyman H., Aslan A., Bayir Y., Halici M., Kazaz C.J. (2006). Gastroprotective and antioxidant effects of usnic acid on indomethacin-induced gastric ulcer in rats. J. Ethnopharmacol..

[16] Dévéhat F., Tomasi S., Elix J.A., Bernard A., Rouaud I., Uriac P., Boustie J. (2007). Stictic Acid Derivatives from the Lichen *Usnea articulata* and Their Antioxidant Activities. J. Nat. Prod..

[17] Behera B.C., Mahadik N., Morey M. (2012). Antioxidative and cardiovascular-protective activities of metabolite usnic acid and psoromic acid produced by lichen species *Usnea complanata* under submerged fermentation. Pharm. Biol..

[18] Santos L.C., Honda N.K., Carlos I.Z., Vilegas W. (2004). Intermediate reactive oxygen and nitrogen from macrophages induced by Brazilian lichens. Fitoterapia.

[19] Ranković B., Kosanić M., Stanojković T., Vasiljević P., Manojlović N. (2012). Biological Activities of *Toninia candida* and *Usnea barbata* Together with Their Norstictic Acid and Usnic Acid Constituents. Int. J. Mol. Sci..

[20] Bessadottir M., Egilsson M., Einarsdottir E., Magnusdottir I.H., Ogmundsdottir M.H., Omarsdottir S., Ogmundsdottir H.M. (2012). Proton-Shuttling Lichen Compound Usnic Acid Affects Mitochondrial and Lysosomal Function in Cancer Cells. PLoS One.

[21] De Paz G.A., Raggio J., Gómez-Serranillos M.P., Palomino O.M., González-Burgos E., Carretero M.E., Crespo A. (2010). HPLC isolation of antioxidant constituents from *Xanthoparmelia* spp. J. Pharm. Biomed. Anal..

[22] Jin J.Q., Li C.Q., He L.C. (2008). Down-regulatory effect of usnic acid on nuclear factor-kappaB-dependent tumor necrosis factor-alpha and inducible nitric oxide synthase expression in lipopolysaccharide-stimulated macrophages RAW 264.7. Phytother. Res..

[23] Brisdelli F., Perilli M., Sellitri D., Piovano M., Garbarino J.A., Nicoletti M., Bozzi A., Amicosante G., Celenza G. (2013). Cytotoxic activity and antioxidant capacity of purified lichen metabolites: An *in vitro* study. Phytother. Res..

[24] Marante F.J.T., Castellano A.G., Rosas F.E., Aguiar J.Q., Barrera J.J.B. (2003). Identification and quantitation of allelochemicals from the lichen *Lethariella canariensis*: Phytotoxicity and antioxidative activity. Chem. Ecol..

[25] Bačkorová M., Bačkor M., Mikeš J., Jendželovský R., Fedoročko P. (2011). Variable responses of different human cancer cells to the lichen compounds parietin, atranorin, usnic acid and gyrophoric acid. Toxicol. In Vitro.

[26] Polat Z., Aydin E., Türkez H., Aslan A. (2013). *In vitro* risk assessment of usnic acid compound. Toxicol. Ind. Health.

[27] Rabelo T.K., Zeidán-Chuliá F., Vasques L.M., Dos Santos J.P., Da Rocha R.F., Pasquali M.A., Rybarczyk-Filho J.L., Araújo A.A., Moreira J.C., Gelain D.P. (2012). Redox characterization of usnic acid and its cytotoxic effect on human neuron-like cells (SH-SY5Y). Toxicol. In Vitro.

[28] Kosanić M., Ranković B., Stanojković T., Rančić A., Manojlović N. (2014). Cladonia lichens and their major metabolites as possible natural antioxidant, antimicrobial and anticancer agents. LWT Food Sci. Technol..

[29] Papadopoulou P., Tzakou O., Vagias C., Kefalas P., Roussis V. (2007). Beta-orcinol metabolites from the lichen *Hypotrachyna revoluta*. Molecules.

[30] Barreto R.S.S., Albuquerque-Júnior R.L.C., Pereira-Filho R.N., Quintans J.S.S., Barreto A.S., DeSantana J.M., Santana-Filho V.J., Santos M.R.V., Bonjardim L.R., Araújo A.A.S. (2013). Evaluation of wound healing activity of atranorin, a lichen secondary metabolite, on rodents. Braz. J. Pharmacogn..

[31] Jayaprakasha G.K., Rao L.J. (2000). Phenolic constituents from the lichen *Parmotrema stuppeum* (Nyl.) Hale and their antioxidant activity. Z. Naturforsch. C.

[32] Lopes T.I., Coelho R.G., Yoshida N.C., Honda N.K. (2008). Radical-scavenging activity of orsellinates. Chem. Pharm. Bull..

[33] Bayir Y., Odabasoglu F., Cakir A., Aslan A., Suleyman H., Halici M., Kazaz C. (2006). The inhibition of gastric mucosal lesion, oxidative stress and neutrophil-infiltration in rats by the lichen constituent diffractaic acid. Phytomedicine.

[34] Liu W., Xu Z., Yang T., Deng Y., Xu B., Feng S., Li Y. (2014). The protective role of tea polyphenols against methylmercury-induced neurotoxic effects in rat cerebral cortex via inhibition of oxidative stress. Free Radic. Res..

[35] Bhullar K.S., Rupasinghe H.P. (2013). Polyphenols: Multipotent Therapeutic Agents in Neurodegenerative Diseases. Oxidative Med. Cell. Longev..

[36] Odabasoglu F., Yildirim O.S., Aygun H., Halici Z., Halici M., Erdogan F., Cadirci E., Cakir A., Okumus Z., Aksakal B. (2012). Diffractaic acid, a novel proapoptotic agent, induces with olive oil both apoptosis and antioxidative systems in Ti-implanted rabbits. Eur. J. Pharmacol..

[37] Bhattarai H.D., Kim T., Oh H., Yim J.H.J. (2013). A new pseudodepsidone from the Antarctic lichen *Stereocaulon alpinum* and its antioxidant, antibacterial activity. J. Antibiot.

[38] Manojlović N., Ranković B., Kosanić M., Vasiljević P., Stanojković T. (2012). Chemical composition of three *Parmelia* lichens and antioxidant, antimicrobial and cytotoxic activities of some their major metabolites. Phytomedicine.

[39] Pavlovic V., Stojanovic I., Jadranin M., Vajs V., Djordjević I., Smelcerovic A., Stojanovic G. (2013). Effect of four lichen acids isolated from *Hypogymnia physodes* on viability of rat thymocytes. Food Chem. Toxicol..

[40] Kosanić M., Manojlović N., Janković S., Stanojković T., Ranković B. (2013). *Evernia prunastri* and *Pseudoevernia furfuraceae* lichens and their major metabolites as antioxidant, antimicrobial and anticancer agents. Food Chem. Toxicol..

[41] Stojanović I., Najman S., Jovanović O., Petrović G., Najdanović J., Vasiljević P., Šmelcerović A. (2014). Effects of Depsidones from *Hypogymnia physodes* on HeLa Cell Viability and Growth. Folia Biol..

[42] Kinoshita K., Togawa T., Hiraishi A., Nakajima Y., Koyama K., Narui T., Wang L.S., Takahashi K. (2010). Antioxidant activity of red pigments from the lichens *Lethariella sernanderi*, *L. cashmeriana*, and *L. sinensis*. J. Nat. Med..

[43] Paudel B., Bhattarai H.D., Koh H.Y., Lee S.G., Han S.J., Lee H.K., Oh H., Shin H.W., Yim H. (2011). Ramalin, a novel nontoxic antioxidant compound from the Antarctic lichen *Ramalina terebrata*. Phytomedicine.

[44] Takenaka Y., Tanahashi T., Nagakura N., Hamada N. (2000). Production of xanthones with free radical scavenging properties, emodin and sclerotiorin by the cultured lichen mycobionts of *Pyrenula japonica*. Z. Naturforsch. C.

[45] Russo A., Piovano M., Lombardo L., Garbarino J., Cardile V. (2008). Lichen metabolites prevent UV light and nitric oxide-mediated plasmid DNA damage and induce apoptosis in human melanoma cells. Life Sci..

[46] Luo H., Li C., Kim J.C., Liu Y., Jung J.S., Koh Y.J., Hur J.S. (2013). Biruloquinone, an Acetylcholinesterase Inhibitor Produced by Lichen-Forming Fungus *Cladonia macilenta*. J. Microbiol. Biotechnol..

[47] Boustie J., Grube M. (2005). Lichens—A promising source of bioactive secondary metabolites. Plant Genet. Resour..

[48] Cowan D.A., Green T.G.A., Wilson A.T. (1979). Lichen metabolism—Aspects of light and dark physiology. New Phytol..

[49] Liberati A., Altman D.G., Tetzlaff J., Mulrow C., Gøtzsche P.C., Ioannidis J.P.A., Clarke M., Devereaux P.J., Kleijnen J., Moher D. (2009). The PRISMA statement for reporting systematic reviews and meta-analyses of studies that evaluate health care interventions: Explanation and elaboration. BMJ.

