# Supplementary material for: Antioxidant Activity and Mechanisms of Action of Natural Compounds Isolated from Lichens: A Systematic Review
Source: Molecules. 2014 Sep 12;19(9):14496–527. doi: 10.3390/molecules190914496 (PMC6271897; doi:10.3390/molecules190914496)
Supplement: Supplementary File 1 [file molecules-19-14496-s001.pdf]

# Supplementary Materials

**Figure S1.** Chemical structures of the compounds isolated from lichens.

| | | |
|---|---|---|
| 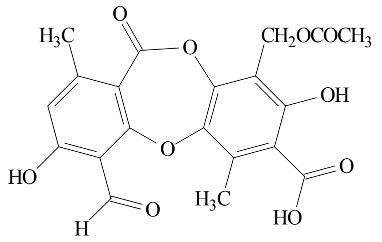 | 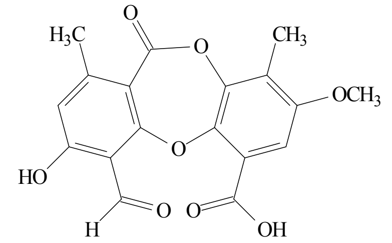 | 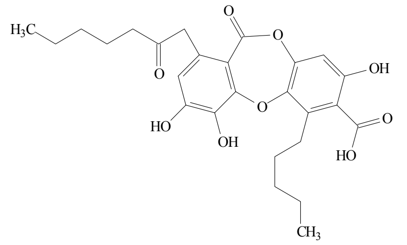 |
| Physodalic acid | Psoromic acid | 3-Hydroxy-physodic acid |
| 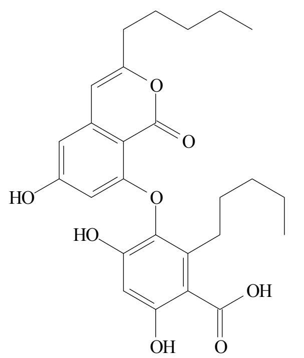 | 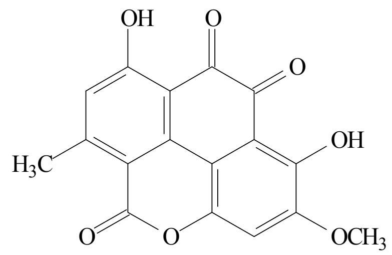 | 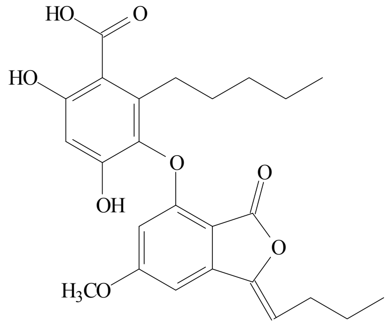 |
| Isophysodic acid | Biruloquinone | Lobastin |
| 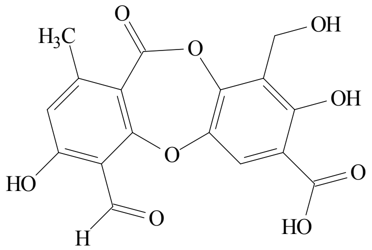 | 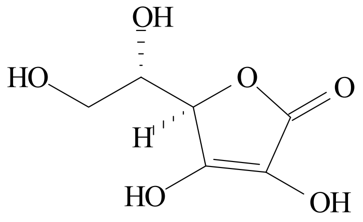 | 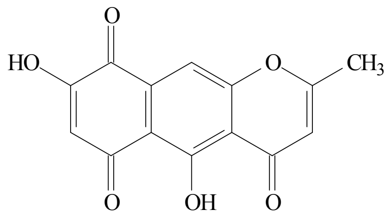 |
| Protocetraric acid | Ascorbic acid | Canarione |
| 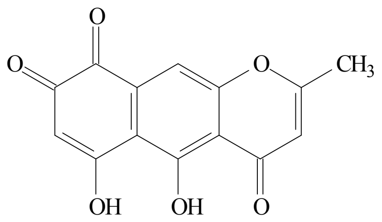 | 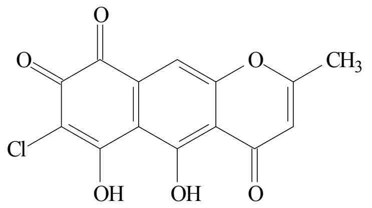 | 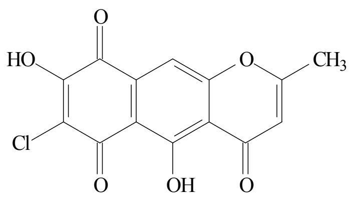 |
| Rubrocashmeriquinone | 7-Chlororubrocashmeriquinone | 7-Chlorocanarione |
| 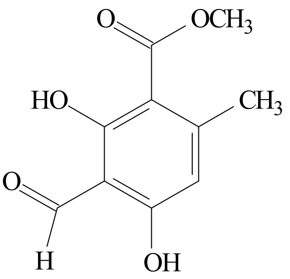 | 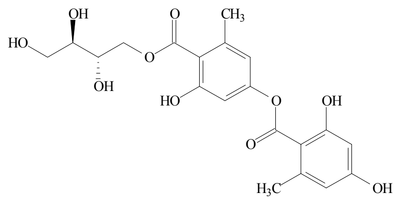 | 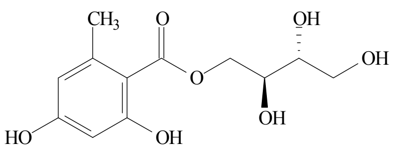 |
| Methyl haematommate | Erythrin | Montagnetol |

**Figure S1.** *Cont.*

| | | |
|---|---|---|
| 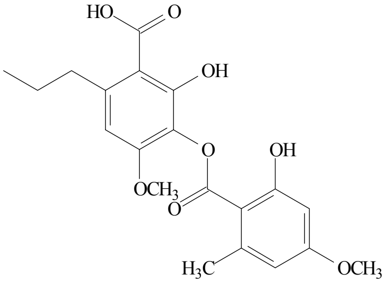 | 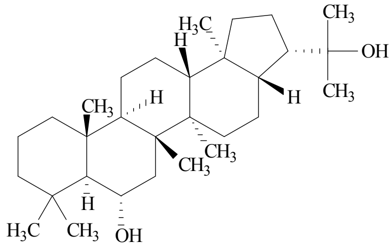 | 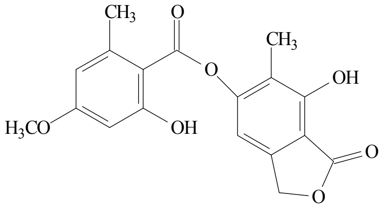 |
| Sekikiac acid | Zeorin | Hypotrachynic acid |
| 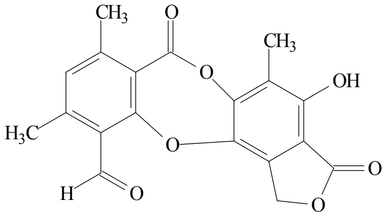 | 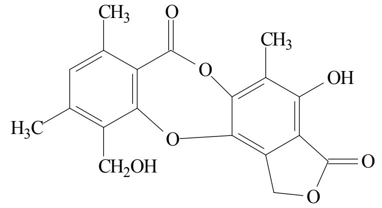 | 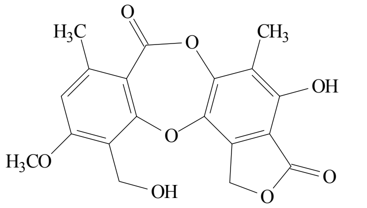 |
| Deoxystictic acid | Cryptostictinolide | Cryptostictinolide |
| 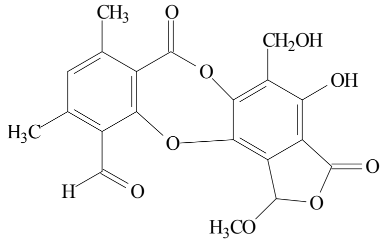 | 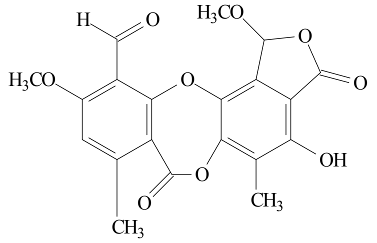 | 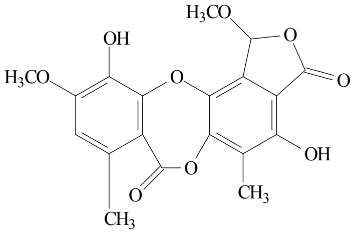 |
| 8'-Methylconstictic acid | 8'-Methylstictic acid | 8'-Methylmenegazziaic acid |
| 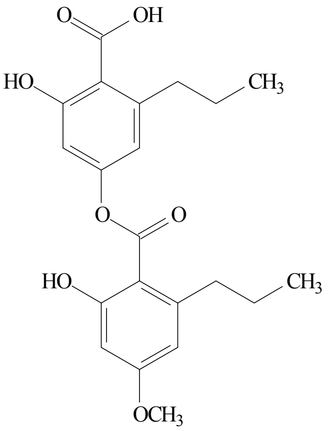 | 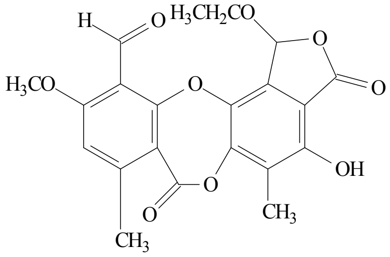 | 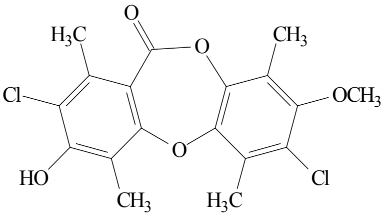 |
| Divaricatic acid | 8'-Ethylstictic acid | Vicanicin |
| 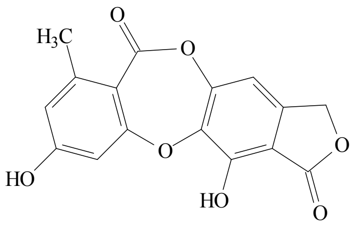 | 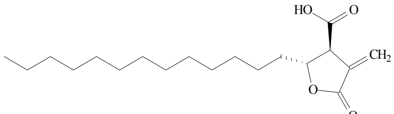 | 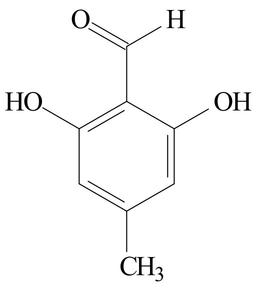 |
| Variolaric acid | Protolichesterinic acid | Atranol |

**Figure S1.** ***Cont.***

| | | |
|---|---|---|
| 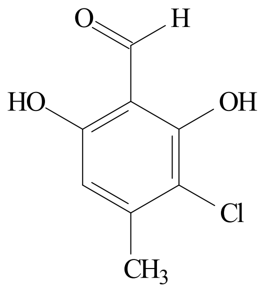 | 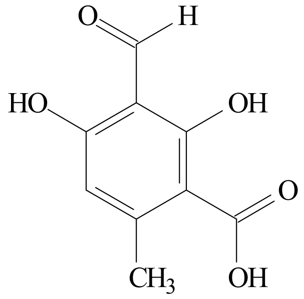 | 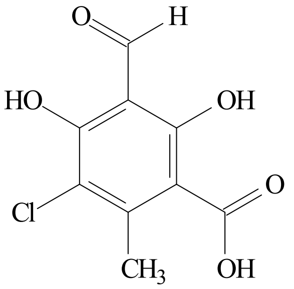 |
| Chloroatranol | Haematommic acid | Chlorohaematommic acid |
| 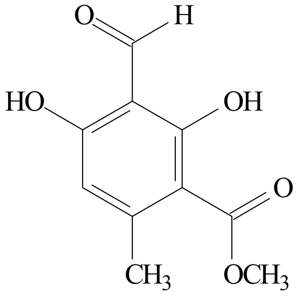 | 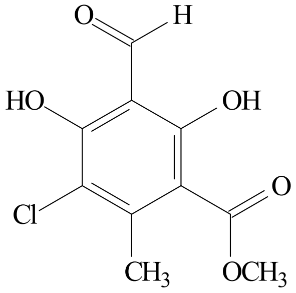 | 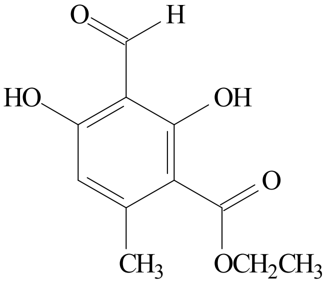 |
| Methyl haematommate | Methyl chlorohaematommate | Ethyl haematommate |
| 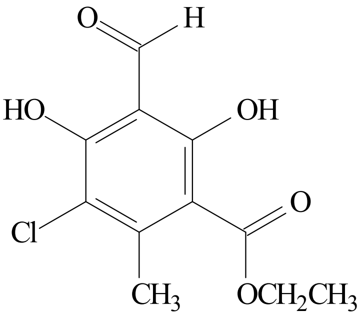 | 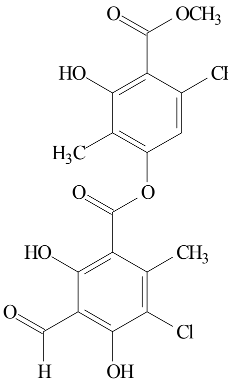 | 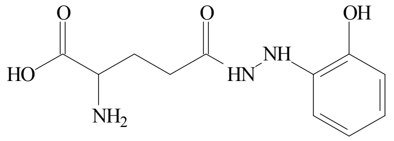 |
| Ethyl chlorohaematommate | Chloroatranorin | Ramalin |
| 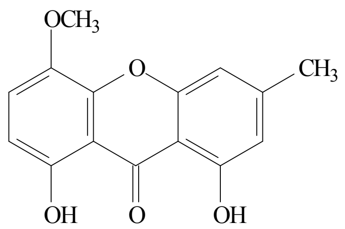 | 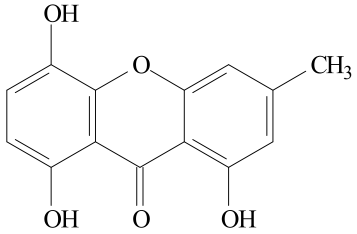 | 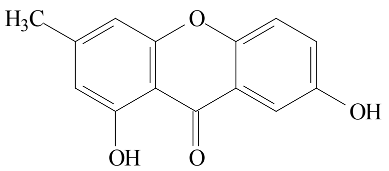 |
| 1,8-Dihydroxy-5-methoxy-3-methylxanthone | 1,5,8-Trihydroxy-3-methylxanthone | 1,7-Dihydroxy-3-methylxanthone |

**Figure S1.** *Cont.*

| | | |
|---|---|---|
| 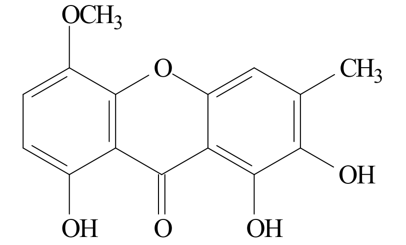 | 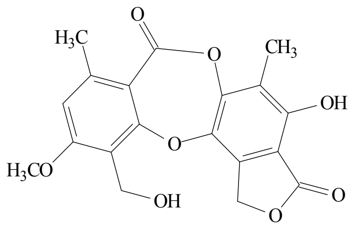 | 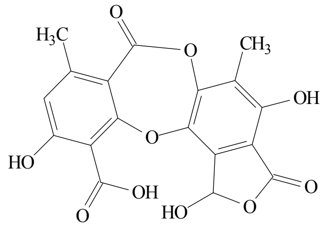 |
| 1,2,8-Trihydroxy-5-methoxy-3-methylxanthone | Cryptostictinolide | Norstictic acid |
| 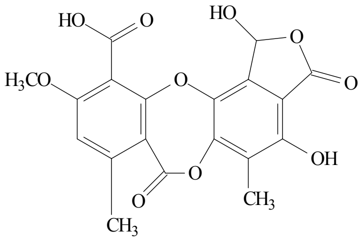 | 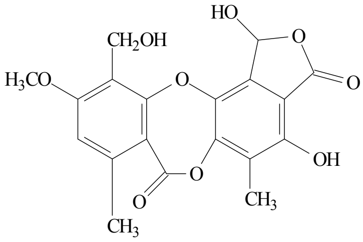 | 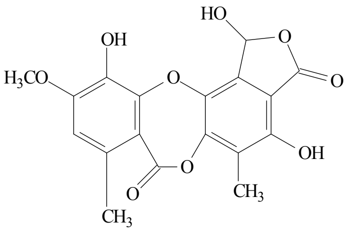 |
| Peristictic acid | Cryptostictic acid | Menegazziaic acid |
| 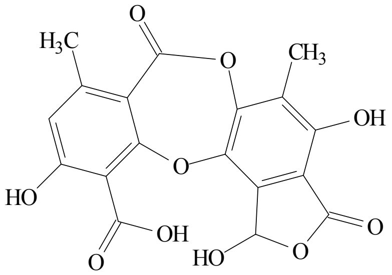 | 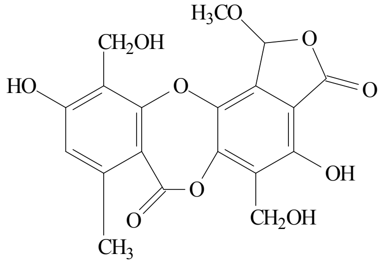 | 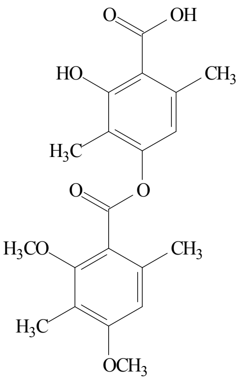 |
| Constictic acid | 3-*O*-Methylconsalazinic acid | Barbatic acid |
| 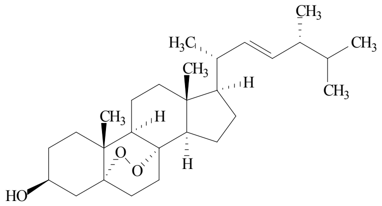 | 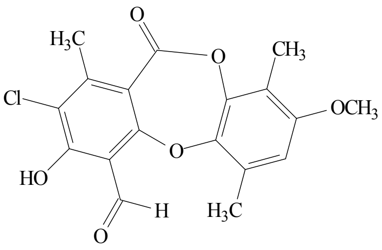 | 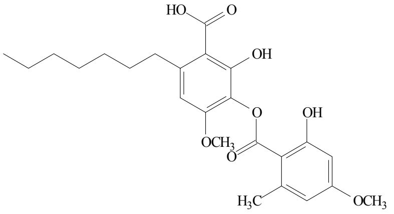 |
| Ergosterol peroxide | Pannarin | Sphaerophorin |
